# Multireader sample size program for diagnostic studies: demonstration and methodology

**DOI:** 10.1117/1.JMI.5.4.045503

**Published:** 2018-11-30

**Authors:** Stephen L. Hillis, Kevin M. Schartz

**Affiliations:** a University of Iowa, Departments of Radiology and Biostatistics, Iowa City, Iowa, United States; b University of Iowa, Department of Radiology, Iowa City, Iowa, United States

**Keywords:** sample size estimation, reader performance, diagnostic radiology, Obuchowski–Rockette, Dorfman–Berbaum–Metz, multireader multicase

## Abstract

The software “Multireader sample size program for diagnostic studies,” written by Kevin Schartz and Stephen Hillis, performs sample size computations for diagnostic reader-performance studies. The program computes the sample size needed to detect a specified difference in a reader-performance measure between two imaging modalities when using the analysis methods initially proposed by Dorfman, Berbaum, and Metz, and Obuchowski and Rockette, and later unified and improved by Hillis and colleagues. A commonly used reader-performance measure is the area under the receiver-operating-characteristic curve. The program has an easy-to-use step-by-step intuitive interface that walks the user through the entry of the needed information. It can be used with several different study designs, inference procedures, hypotheses, and input and output formats. The program is functional in Windows, OS X, and Linux. The methodology underlying the software is discussed for the most common diagnostic study design, where each reader evaluates each case using each modality.

## 1 Introduction

One of the most important aspects of designing a diagnostic reader-performance study is determination of adequate sample size. The sample size should be large enough to provide sufficient power (typically at least 0.80) to detect a specified effect size, which often is defined as the difference in population-averaged reader-performance measures between two imaging modalities. Ideally, the specified effect size represents the minimum effect size that is considered to be clinically significant, but because of financial or logistical considerations the researcher may specify a somewhat larger effect size. In addition, prior information can influence the selection of the specified effect size. In Sec. 4, we discuss selection of the predetermined effect size in more detail.

The specified effect size should be determined by the researcher in the planning stage of the study. The topic of this paper is computation of adequate sample size for diagnostic radiologic studies involving several readers (typically radiologists), which we refer to as multireader multicase (MRMC) studies, for a specified effect size.

A typical MRMC study design is the factorial design, where each case (i.e., patient) undergoes each of several diagnostic tests (or imaging modalities) and the resulting images are interpreted by each of several readers. Often each reader is asked to assign a confidence-of-disease rating to each case for each test, based on the corresponding image or set of images, and a receiver-operating-characteristic (ROC) curve for each reader is estimated from the case-level ratings. The diagnostic tests are then compared with respect to reader-performance outcomes that are typically functions of the reader ROC curves. A commonly used reader-performance summary outcome is the area under the ROC curve (AUC). Usually, it is desired that conclusions generalize to both the reader and case (i.e., patient) populations, rather than to only one of the populations.

This paper serves two purposes: (1) It provides an introductory demonstration of the recently created software program “Multireader sample size program for diagnostic studies” (freely available in Ref. 1), written by the authors, for sizing MRMC studies. (2) It discusses the statistical methodology underlying the software, providing sufficient information for readers who may want to create their own sample size software or to include sample size estimation in simulation studies. The introductory software demonstration requires only a basic understanding of statistics, comparable to that provided in an introductory statistics course. In contrast, the discussion of the underlying statistical methodology requires a higher level of understanding, comparable to that gained from taking statistics courses in mathematical statistics, regression, and design of the experiments. However, we emphasize that use of the software does not require extensive statistical methodology understanding; thus for readers only interested in using the software to size studies, we suggest either skimming or skipping Sec. 7, which contains the discussion of the underlying statistical methodology.

The software is based on the Obuchowski and Rockette (OR)^2^ and Dorfman, Berbaum, and Metz (DBM)^3^^,^^4^ analysis methods, which are the most frequently used methods for analyzing MRMC studies. The OR method includes the DBM method as a special case, and hence is the more general method. Since the OR and DBM methods were first proposed, they have been unified, improved, and generalized by Hillis and colleagues.^5^^–^^8^ It is these improved versions of OR and DBM, which are what are used today, that the program is based on.

The program computes the reader and case sample sizes needed to detect a specified effect size, defined as the difference in a reader-performance measure (frequently AUC) between two tests. The sample size computations depend on the user-specified effect size and on the user-supplied parameter values that describe the distribution of the reader performance outcomes. The needed parameter value inputs can be estimated from pilot data or from previous similar studies or can be conjectured.

Important contributions of the software are the following: (1) In addition to the factorial design, the software can be used for four other study designs. (2) Although usually it is desirable to generalize to both the reader and case populations, sometimes an analysis that generalizes only to the reader or case populations may be more appropriate because of the study design or limited resources. This software can be used for any of these three inference situations.

An outline of the paper is as follows. In Sec. 2, we discuss features of the program and in Sec. 3 we present illustrative examples of running the program using pilot-data parameter estimates. In Sec. 4, we discuss specification of the effect size, in Sec. 5, we discuss using the software with designs other than the factorial design, and in Sec. 6, we provide an introduction to determining conjectured values. Methodology underlying the program is discussed in Sec. 7 for the most commonly used study design, the factorial design, where each reader evaluates each case using under each test. We emphasize that Sec. 7 can be skipped for readers primarily interested in using the software. Concluding remarks are made in Sec. 8.

## 2 Features of the Program

### 2.1 Functionality

The program file is an executable Java jar file that runs on Windows, OS X, and Linux. The same downloadable file can be used with all three operating systems.

### 2.2 Outcomes

The program can be used with typical reader-performance measures, such as sensitivity, specificity, and ROC curve summary measures, which include AUC, partial AUC, sensitivity for specified specificity, and specificity for specified sensitivity. These measurements can be estimated using parametric or nonparametric methods. In addition, the program can be used with free-response ROC (FROC),^9^^,^^10^ localization-response operating characteristic (LROC),^11^^–^^13^ and region-of-interest (ROI)^14^ summary measures. For simplicity, we often implicitly assume that the reader-performance measure of interest is AUC.

### 2.3 OR and DBM Inputs: Input Conversion Program

For the factorial study design, the DBM method is equivalent to the OR method when both use the same AUC estimation method and OR uses the jackknife method for estimating the error variance and covariances (due to reading the same cases). The OR method is more general than DBM because it can accommodate other methods of estimating the error covariances, such as the method of DeLong et al.^15^ for trapezoid AUC estimates and the method of bootstrapping. The program allows the user to perform analyses based on output from either OR or DBM analyses. Although the program will ask the user for OR parameters values, the “input conversion program” that is available from the help menu can be used to convert DBM parameter values, DBM mean squares, and OR mean squares to the needed OR parameter values.

One reason we emphasize the OR parameter values over the DBM parameter values is because they are easier to interpret. Another reason is that the OR model, because of its firmer statistical foundation, has been the basis for all new development in the last 10 years. For example, although the OR approach has been developed^8^ for all five of the study designs included in this software, the DBM approach has only been developed for the factorial design.

### 2.4 Inference Situations

The program computes sample sizes for three inference situations:

1. Both readers and cases are random.
2. Readers are fixed and cases are random.
3. Readers are random and cases are fixed.

Corresponding analysis results generalize, respectively, to (1) the reader and case populations for which the study reader and cases are representative; (2) the case population when evaluated by the particular readers in the study; and (3) the reader population when evaluating the particular cases used in the study. Determination of the appropriate inference situation depends on the research question and study design.

Researchers typically would like to generalize to both the reader and case populations, which requires a study having more than one reader in order to estimate between-reader variability. Although theoretically such a study can have as few as two or three readers, results are more convincing with at least four or five readers since then the sample seems more likely to be representative of a population of similar readers. Thus, we recommend that a researcher use at least four readers, preferably more, if the goal is to generalize to both reader and case populations. If financial or logistical concerns limit the number of readers to less than four, then we recommend using a fixed-readers and random-cases (inference situation 2) analysis. Even though such a study does not generalize to readers, it can provide an important first step in establishing a conclusion (e.g., one modality is superior when used by the readers in the study) when previous studies have not been undertaken.

A random-readers and fixed cases (inference situation 3) analysis may be appropriate when an inference situation 1 analysis is not feasible, especially when generalization to the reader population is deemed more important than to the case population. This can happen, e.g., when the “test” factor is a reader psychological or demographic factor. For instance, a researcher may want to compare performance for radiologists versus residents or for readers grouped according to how many cases they read per year. For these examples, each test level represents a descriptive quality of the reader, and hence each reader evaluates cases under only one of the test levels. These are examples of the reader-nested-within-test split plot study design, discussed in Sec. 5. An alternative analysis approach for this design is the nonparametric Wilcoxon rank-sum test, which similarly gives conclusions that generalize to the respective reader populations when restricted to reading the study cases. In practice, such comparisons are often secondary comparisons performed on data resulting from a factorial study where the primary aim is to compare imaging modalities. For example, each reader reads each case under each modality and the modalities are compared using the type 1 inference approach, but the researcher also wants to compare residents versus radiologists within each modality (a reader-nested-wthin-test analysis) using a type 3 inference analysis to increase power.

Regardless of what analysis method is used, it is important that authors state for which populations (reader, case, or both reader and case) conclusions are applicable and discuss the rationale for the analysis (e.g., why was an analysis chosen that generalizes to only one population instead of both populations). Unfortunately, all too often this information is not provided, making it difficult for the reader to discern the scope of the conclusions.

### 2.5 Hypotheses Tests

Either nonequivalence or noninferiority alternative hypotheses tests can be specified. Both hypotheses are defined in terms of expected reader performance outcomes. Statistical details for both types of tests are provided in Sec. 7.2. The program only allows for the comparison of two modalities.

### 2.6 Obtaining Input Values from Pilot Data

Pilot data estimates can be obtained from OR or DBM analyses. Pilot data estimates from a factorial-design study can be used as inputs for all of the designs. Software for performing the OR and DBM methods for ROC data is freely available in Ref. 1 in both a stand-alone version and in a version designed to be run with SAS statistical software. For OR and DBM analyses of FROC and ROI data, freely available stand-alone software is available in Ref. 16.

### 2.7 User Manual

A user manual can be accessed from the help menu. It provides illustrative examples of different sample-size analyses.

### 2.8 Running the Program

The program is designed with an intuitive point-and-click interface. In the next section, we provide several examples illustrating use of the program.

## 3 Examples of Running the Program Using OR Inputs

### 3.1 Pilot Data

To illustrate use of the program, we treat study data provided by Carolyn Van Dyke, MD (Van Dyke)^17^ as pilot data for sizing a future study. The Van Dyke study compares the relative performance of single spin-echo magnetic resonance imaging (MRI) to cinematic presentation of MRI for the detection of thoracic aortic dissection. There are 45 patients with an aortic dissection and 69 patients without a dissection imaged with both spin-echo and cinematic MRI. In this factorial-design study, five radiologists independently interpret all of the images using a five-point ordinal scale: 1 = definitely no aortic dissection,…, 5 = definitely aortic dissection. These data are available in Ref. 1.

For this study, the average spin-echo empirical AUC is 0.044 larger than the average cine empirical AUC (spin-echo average = 0.941, cine average = 0.897); however, there is not a significant difference (p=0.0517) between the modalities based on either a DBM or the equivalent OR analysis using jackknife error covariance estimation. The 95% confidence interval (CI) for the (spin-echo minus cine) difference in the reader-averaged modality AUCs is (−0.00036,0.088). For the examples in Secs. 3.4–3.7, we consider the situation where a researcher would like to know what combinations of reader and case sample sizes for a similar study will have at least 0.80 power to detect an absolute difference of 0.05 between the modality AUCs. We show how to determine the smallest case sample size for each of several reader sample sizes that yields 0.80 power for detecting a 0.05 difference in spin-echo and cinematic AUC, based on parameter estimates computed from the Van Dyke data. We set alpha, the probability of a type I error, equal to 0.05. In Sec. 3.8, we show how to compute the needed sample sizes for testing if one modality is noninferior to the other.

### 3.2 Obuchowski–Rockette Model

Let ${\hat{\theta }}_{ij}$ denote the AUC estimate for reader j using test i. For analyzing these reader performance outcomes, OR^2^ proposed the following ANOVA model where the error terms are correlated to account for correlation resulting from each reader evaluating the same cases:

Model M1:  θ^ij=μ+τi+Rj+(τR)ij+ϵij, (1)

where μ is the fixed intercept term, $\tau_{i}$ denotes the fixed effect of test i, $R_{j}$ denotes the random effect of reader j, ${\left(\tau R\right)}_{ij}$ denotes the random test-by-reader interaction, and $\epsilon_{ij}$ is the random error term. All random effects are normally distributed with zero means. We let $\sigma_{R}^{2}$, $\sigma_{TR}^{2}$, and $\sigma_{\epsilon }^{2}$ denote the variances of the reader, test-by-reader, and error random effects, respectively. Model M1 treats both reader and case as random factors, and thus conclusions generalize to both the reader and case populations.

Equicovariance of the errors between readers and tests is assumed, resulting in three possible covariances:

$$\mathrm{Cov}(\epsilon_{ij},\epsilon_{i^{\prime }j^{\prime }})=\{\begin{array}{ll} {\mathrm{Cov}}_{1} & i\neq i^{\prime },j=j^{\prime }(\text{different test},\text{same reader})\\ {\mathrm{Cov}}_{2} & i=i^{\prime },j\neq j^{\prime }(\text{same test},\text{different reader})\\ {\mathrm{Cov}}_{3} & i\neq i^{\prime },j\neq j^{\prime }(\text{different test},\text{different reader}) \end{array}.$$ (2)

We assume

$${\mathrm{Cov}}_{1}\geq {\mathrm{Cov}}_{3},\;{\mathrm{Cov}}_{2}\geq {\mathrm{Cov}}_{3}\;\text{and}\;{\mathrm{Cov}}_{3}\geq 0$$ (3)

as recommended by Hillis.^8^ The quantities $\sigma_{\epsilon }^{2}$, ${\mathrm{Cov}}_{1}$, ${\mathrm{Cov}}_{2}$, and ${\mathrm{Cov}}_{3}$ are typically estimated using the jackknife,^18^ bootstrap,^19^ or the method of DeLong et al.^15^ Model M1 can alternatively be described with population correlations

$$r_{i}={\mathrm{Cov}}_{i}/\sigma_{\epsilon }^{2},\;i=1,2,3$$ (4)

instead of the covariances, i.e., with ${\mathrm{Cov}}_{i}$ replaced by $r_{i}\sigma_{\epsilon }^{2}$, i=1,2,3. (See Sec. 7 for a more detailed discussion of the OR model.)

### 3.3 Parameter Estimates from Pilot Data

**Fig. 1:**
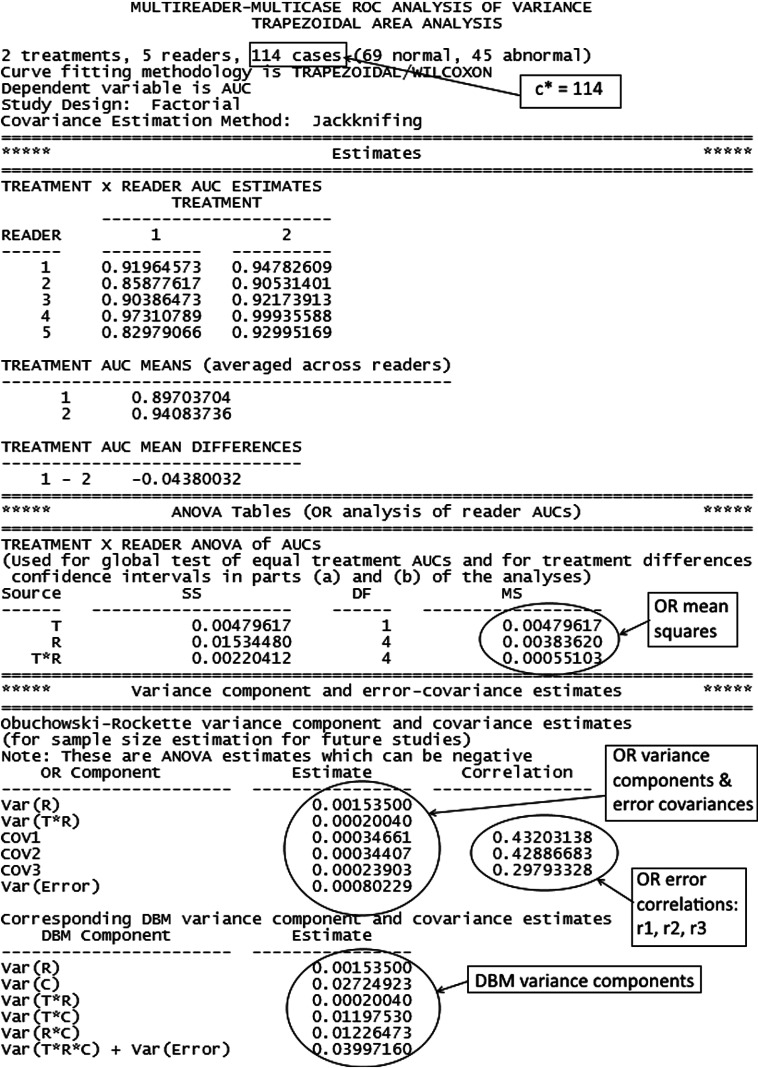
Partial OR output from ROC AUC analysis of Van Dyke^17^ data using OR-DBM MRMC 2.5 software.

Partial output from performing an OR analysis comparing empirical AUCs using OR-DBM MRMC 2.5 software (available in Ref. 20) with jackknife covariance estimation is presented in Fig. 1. In Fig. 1, the “estimates” section presents the reader AUC estimates and the “ANOVA tables (OR analysis of reader AUCS)” section presents the ANOVA table corresponding to the OR method. The “variance component and error-covariance estimates” section presents the OR variance components and error covariance estimates in the upper half, and the DBM variance components estimates in the lower half for readers familiar with the DBM method. For the sample size program only OR estimates are required, and thus only these will be discussed. The OR variance components and error covariances, OR error correlations and OR mean squares variance components are labeled in Fig. 1, with “Var(R),” “Var(T*R),” and “Var(Error)” denoting the reader, test-by-reader, and error random effect variances (i.e., $\sigma_{R}^{2}$, $\sigma_{TR}^{2}$, and $\sigma_{\epsilon }^{2}$) for model M1. Figure 1 provides all the needed information for performing sample size estimation for a future study. Although the parameter estimates in Fig. 1 are from a factorial study, these estimates can also be used as inputs for any of the other four study designs, as will be discussed in Sec. 5.2.

### 3.4 Example: Running the Sample-Size Program for Random Readers and Cases

We now show how to run the program for an analysis that treats both readers and cases as random.

**Fig. 2:**
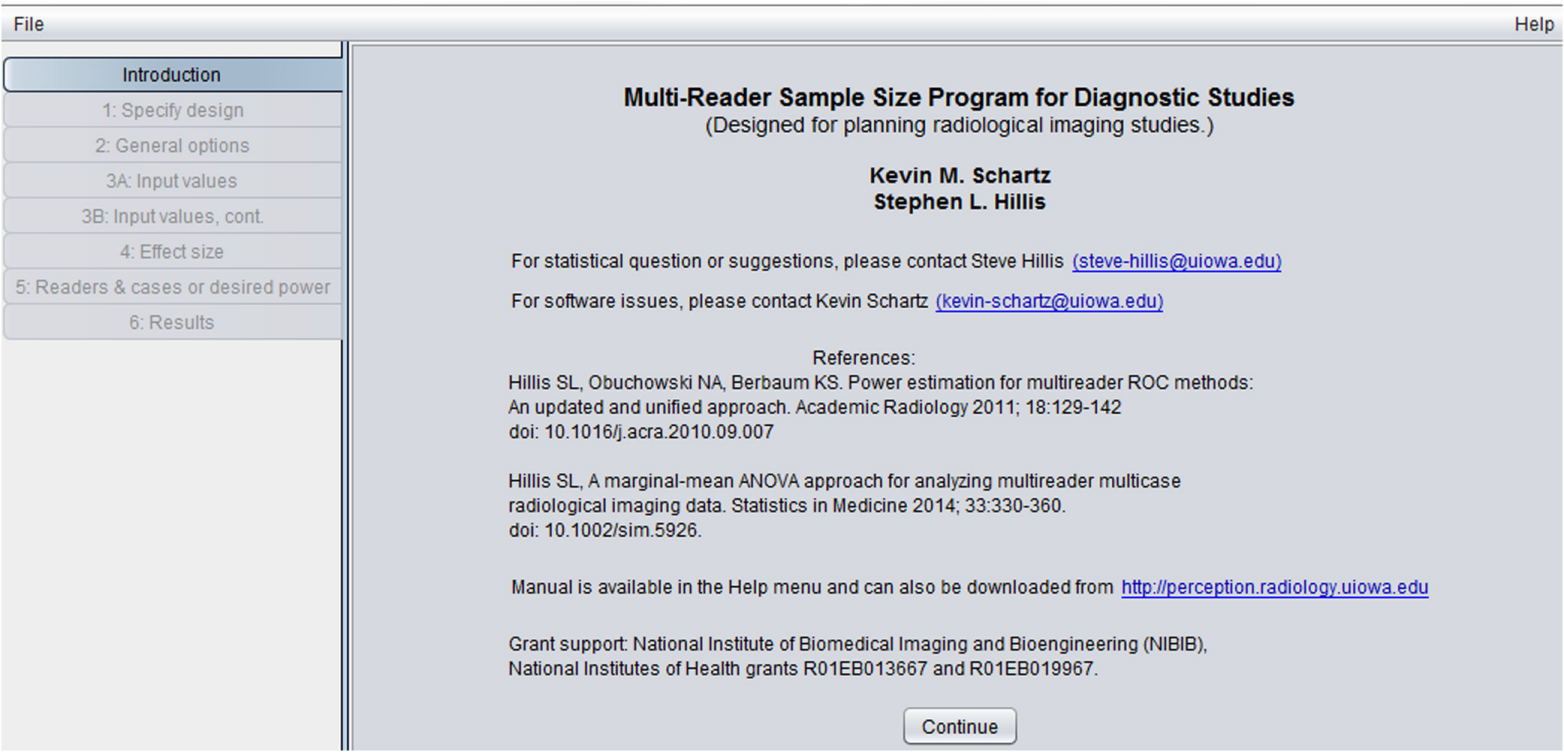
First window in sample-size program.

The first window of the program is shown in Fig. 2. This window provides contact information for questions and references that the software is based on.

**Fig. 3:**
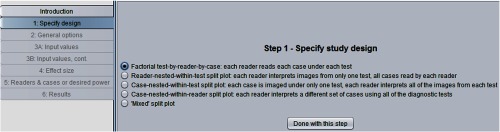
Step 1 in sample-size program.

Figure 3 shows the “step 1: specify study design” window. Here, we have indicated that we want to do sample-size estimation for a factorial study. Note that any of the four other designs, which will be discussed in Sec. 5, could have been selected.

**Fig. 4:**
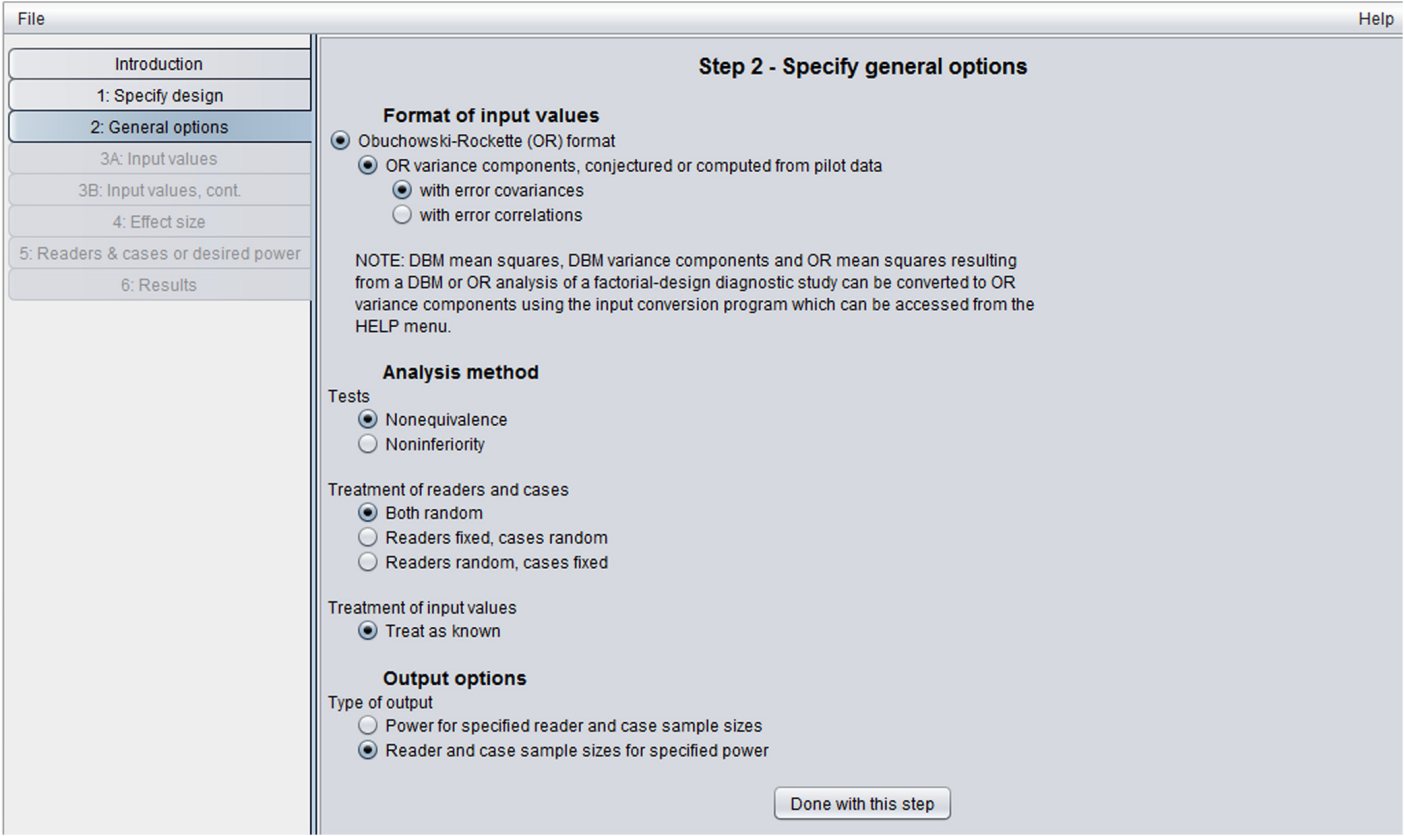
Step 2 in sample-size program.

Figure 4 shows the “step 2: specify general options” window. Here, we have indicated that we will input OR variance components, and we have also chosen to input error covariances rather than error correlations. If these OR parameter values are not available but either (1) OR mean squares and error covariances and variance, (2) DBM variance components, or (3) DBM mean squares are available, then the input conversion program (available in the help menu) can be used to convert these values to the OR input values requested in step 2.

In Fig. 4, we have requested a nonequivalence test and have requested that readers and cases be treated as random so that conclusions will generalize to both the reader and case populations. We have also requested output that includes various combinations of reader and case sample sizes that will result in a specified power (in step 5 we will specify power to be 0.8).

**Fig. 5:**
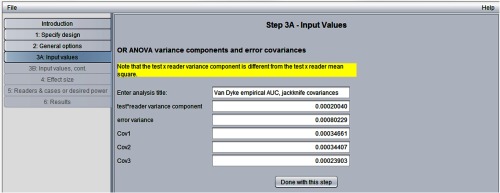
Step 3A in sample-size program.

Figure 5 shows the “step 3A: input values” window. After entering a descriptive file title, we have entered the OR test-by-reader variance component [“Var(T*R)”], error variance [“Var(Error)”], and Cov1, Cov2, and Cov3 values from Fig. 1.

**Fig. 6:**
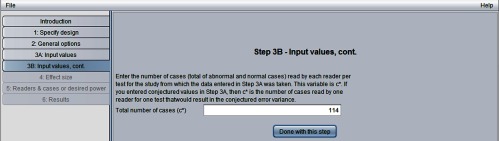
Step 3B in sample-size program.

Figure 6 shows the “step 3B: input values, cont.” window. Here we have entered c* = 114, the number of cases in the Van Dyke study, from Fig. 1.

**Fig. 7:**
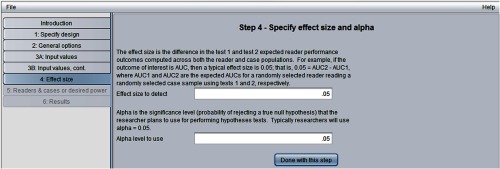
Step 4 in sample-size program.

Figure 7 shows the “step 4: specify effect size and alpha” window. Here, we have indicated the effect size to be an AUC difference of 0.05 and have set alpha equal to 0.05.

**Fig. 8:**
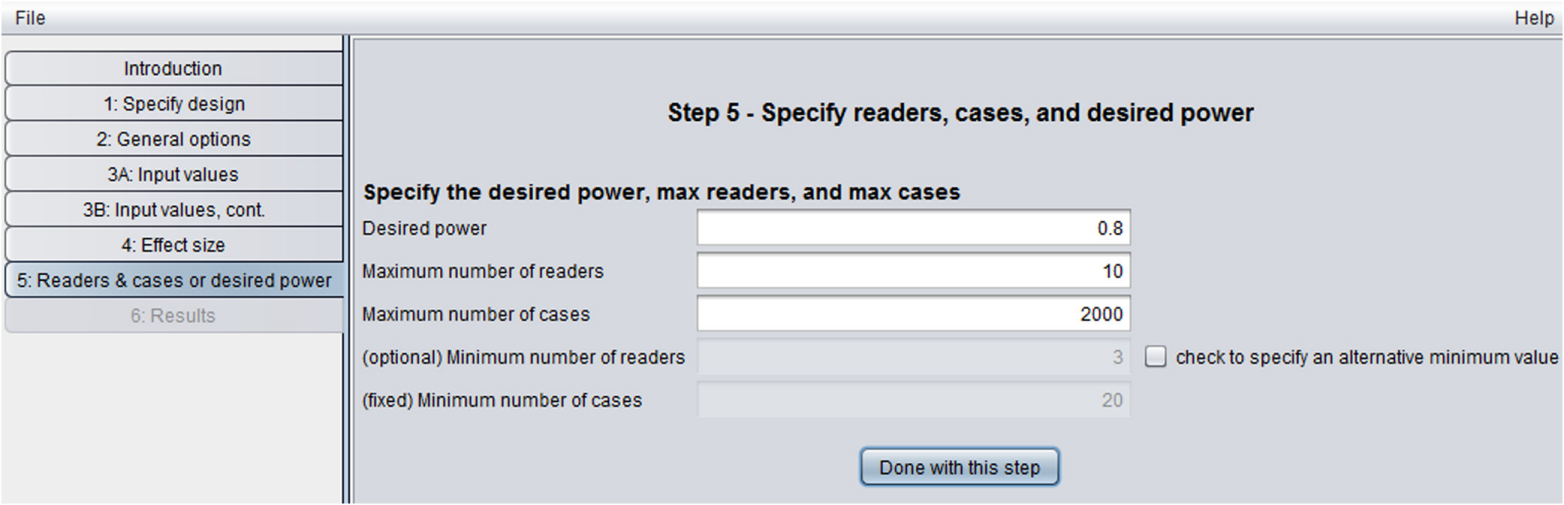
Step 5 in sample-size program.

Figure 8 shows the “step 5: specify readers, cases, and desired power” window. Here, we have requested power = 0.8, and have indicated that the program should compute the number of cases needed for between 3 (the default minimum) and 10 readers, with a maximum of 2000 cases.

**Fig. 9:**
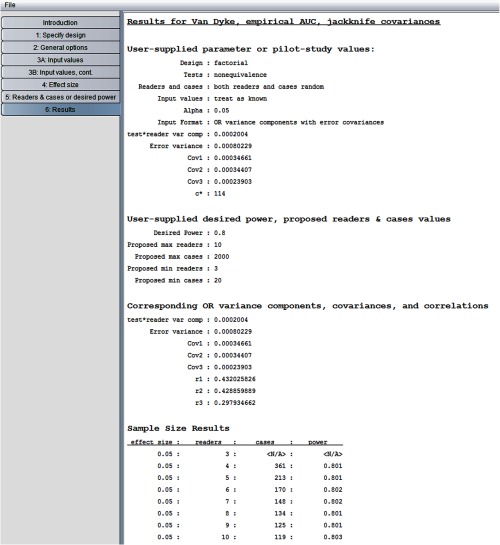
Van Dyke results for random readers, random cases.

Figure 9 shows the “results” window. The window lists user-supplied values in the first two sections. These two sections are followed by the “corresponding OR variance components, covariance, and correlations” section; we previously supplied all of the values in this section except for the error correlations (r1, r2, r3). The “sample size results” section shows the number of cases needed to yield 0.80 power as the number of readers varies between 3 and 10. For example, we see that with six readers we need 170 cases and with five readers we need 213 cases. We see that for three readers the number of cases needed was not less than the specified maximum of 2000, as indicated by “<N/A>.”

### 3.5 Abnormal-to-Normal Case Ratio

Note that the program did not ask for the ratio of abnormal to normal cases, but rather only for the total number of cases for the pilot data. This is because the sample size results assume the same abnormal-to-normal case ratio as for the pilot data, which for the Van Dyke data is 45:69. Thus, for the solution, five readers and 213 cases given in the “sample size results” section in Fig. 9, the 45:69 ratio implies 84 diseased and 129 nondiseased cases. For the situation where the pilot sample ratio is much different from that of the planned study, Hillis et al.^21^ have proposed one way to compute pilot-study estimates that correspond to an abnormal-to-normal case ratio different than that of the pilot study. However, this approach requires resampling and hence is not included in the program.

### 3.6 Fixed Readers Example

For comparison, we rerun the program using the Van Dyke parameter estimates but now treat readers as fixed. The fixed readers OR model is similar to model M1, except that the reader and test-by-reader effects are fixed rather than random, and hence there are no reader and test-by-reader variance parameters. (See Sec. 7.1.2 for a more detailed discussion of this model.) The only change that needs to be made is to request “readers fixed, cases random” instead of “both random” in step 2. In step 5, we again set the maximum number of readers equal to 10.

**Fig. 10:**
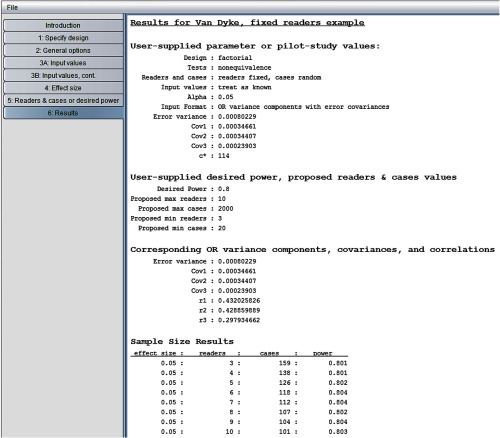
Van Dyke results for fixed readers, random cases.

The resulting “sample size results” window is shown in Fig. 10. We see that considerably fewer cases are required than when readers were treated as random. For example, now 126 cases are needed with five readers, whereas 213 cases were required when readers were treated as random. This not surprising because between-reader variability is not taken into account with fixed readers. Accordingly, step 3 does not ask the user to enter a value for the test-by-reader variance component, which is why it is not listed in Fig. 10.

### 3.7 Fixed Cases Example

**Fig. 11:**
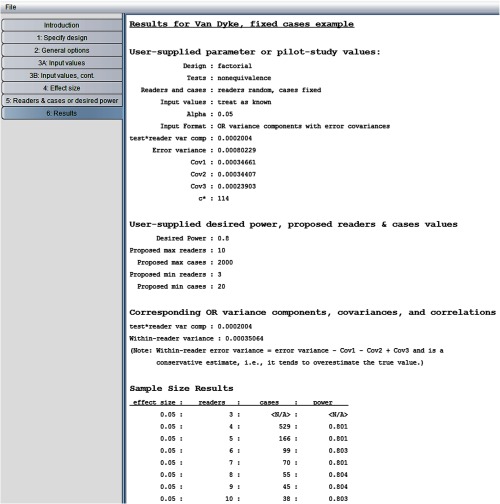
Van Dyke results for random readers, fixed cases.

Now we rerun the program treating cases as fixed. The fixed cases OR model is similar to model M1, except that the error terms are assumed to be independent (see Sec. 7.1.3 for a more detailed discussion of this model). The only change that needs to be made is to request “readers random, cases fixed” in step 2. The resulting “sample size results” window is shown in Fig. 11. We see that 166 cases are needed with five readers, compared to 213 cases when both readers and cases were treated as random. This decrease can be explained by the fact that between-case variability is not taken into account with fixed cases.

Note in Fig. 11 the following statement: “within-reader error variance = error variance - Cov1 − Cov2 + Cov3 and is a conservative estimate, i.e., it tends to overestimate the true value.” Briefly, because cases are fixed, the error variance will be less than for random cases because now it is attributed only to within-reader variability. Without replications (e.g., each reader evaluates each case twice, with a memory washout period separating evaluations), this within-reader error variance cannot be consistently estimated. However, we can estimate an upper bound on it, which is a function of the error variance and covariances for the random-readers and random-cases model, as indicated by the above statement and discussed in more detail in Sec. 7. Thus for this inference situation, the error variance and covariances, obtained from the pilot data from Fig. 1, treating cases as random, are used to estimate the upper bound, which is used as a conservative estimate of the error variance for the fixed-cases computations. Note that although we use the error variance and covariances from model M1 to estimate the error variance for the fixed cases model, the error terms in the fixed-cases model are independent, which implies that the error covariances are all zero.

### 3.8 Noninferiority Hypotheses Example

In Sec. 3.4, we showed how to compute the needed sample size for showing that two modalities are not equivalent. Now suppose instead that our interest is to show that a “new” test is at least as effective as a “standard” test in the sense that if the standard test results in higher performance than the new test, it is by less than a specified amount. Specifically, we want to show that ${\mathrm{AUC}}_{S}$ is less than ${\mathrm{AUC}}_{N}+M$, where ${\mathrm{AUC}}_{S}$ and ${\mathrm{AUC}}_{N}$ are the expected AUCs corresponding to the standard and new tests and M>0 is the noninferiority margin. For this situation, we perform a one-sided noninferiority test, where the null hypothesis is that the new test is inferior to the standard test ($H_{0}:\;{\mathrm{AUC}}_{S}-{\mathrm{AUC}}_{N}\geq M$) and the alternative hypothesis is that it is not inferior ($H_{0}:{\mathrm{AUC}}_{S}-{\mathrm{AUC}}_{N}<M$). These hypotheses and corresponding test are discussed in more detail in Sec. 7.6. Here, we are assuming that a higher reader performance outcome value is indicative of improved performance.

The effect size for the noninferiority test is ${\mathrm{AUC}}_{N}-{\mathrm{AUC}}_{S}$. For example, if it is zero then we are computing the power to conclude that the new test is not inferior to the standard test given that the standard and new tests have the same expected AUC. The effect size does not have to be positive, but it must exceed −M because an effect size ≤−M implies the null hypothesis is true.

**Fig. 12:**
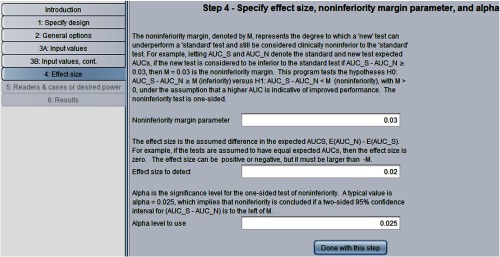
Step 4 for testing noninferiority hypotheses.

For illustration purposes, we now show how to determine the needed numbers of readers and cases to test these noninferiority hypotheses based on the Van Dyke estimates. We rerun the program, again treating both readers and cases as random as in Sec. 3.4. In step 2, we request “noninferiority” test. In step 4, as shown in Fig. 12, we specify the effect size to be 0.02, the inferiority margin to be M=0.03 and α=0.025.

**Fig. 13:**
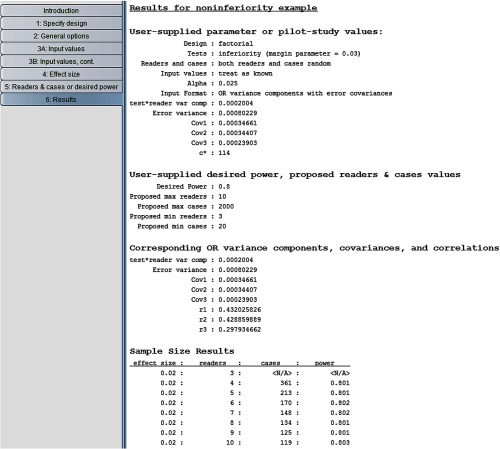
Van Dyke noninferiority results for random readers, random cases.

The results are shown in Fig. 13. Note that these noninferiority-test sample size results with α=0.025, effect size=0.02, and M=0.03 are the same as those in Fig. 9 for the nonequivalence test with α=0.05 and effect size = 0.05. This is because, as will be discussed in Sec. 7.6, for typical power values (e.g., ≥0.70) the noninferiority-test sample size results are essentially the same as for the nonequivalence test with significance level 2α but with the nonequivalence effect size defined as the noninferiority effect size plus M (i.e., 0.02+0.03=0.05).

## 4 Effect Size: Further Considerations

Specification of the effect size for computing power is an important part of sample size estimation. For a detailed treatment of the subject, we recommend the paper by Lenth.^22^ Although it is beyond the scope of this article to give more than a cursory treatment of the subject, below we offer some basic guidelines. In addition, we discuss how to report effect size estimates based on the study data. Throughout this section, we assume that we will perform a nonequivalence test.

### 4.1 Reporting the Estimated Effect Size

After the study has been completed and the test performed, we recommend reporting a 95% CI for the true effect size, as opposed to merely stating whether or not there was enough evidence to conclude the alternative hypothesis. Effect sizes that are contained within a 95% CI are considered to be commensurate with the data, as they cannot be rejected at the 0.05 alpha level using a two-sided hypotheses test; similarly, those values outside of the CI are not considered to be commensurate with the data since they can be rejected. Hoenig and Heisey^23^ point out that “once we have constructed a CI, power calculations yield no additional insights.”

The situation that the researcher wants to avoid is having an insignificant test where some of the effect sizes in the 95% CI are considered to be clinically significant, making it impossible to conclude whether the true effect size is clinically negligible or clinically significant. (Note: by “clinically neglibible” we mean not clinically significant, which is not the same as saying that the effect size is zero.) For example, the analysis of the pilot data discussed in Sec. 3.1 yielded a nonsignificant test result with a point estimate and 95% CI for the effect size given by 0.044 and (−0.00036,0.088), respectively. If the researcher considers an AUC difference of, e.g., 0.08 to be clinically significant, then the researcher can neither conclude that the true effect size is clinically negligible or clinically significant.

Eng^24^ describes the situation succinctly:

In conjunction with a well-defined research question [Eng & Siegelman, 1997], an adequate sample size can help ensure an academically interesting result, whether or not a statistically significant difference is eventually found in the study. The investigator does not have to be overly concerned that the study will only be interesting (and worth the expenditure of resources) if its results are “positive.” For example, suppose a study is conducted to see if a new imaging technique is better than the conventional one. Obviously, the study would be interesting if a statistically significant difference was found between the two techniques. But if no statistically significant difference is found, an adequate sample size allows the investigator to conclude that no clinically important difference was found rather than wonder whether an important difference is being hidden by an inadequate sample size.

### 4.2 Determining the Effect Size for Computing Power

In practice, deciding on a specified effect size is typically an iterative procedure involving three steps: (1) The researcher specifies a minimum effect size for which the researcher wants there to be a specified power to reject the null hypothesis. If this is the first iteration, we recommend the researcher specify what she considers to be the minimum clinically relevant effect size. (2) Reader and case samples are determined that provide the desired power for the specified effect size. (3) The researcher considers whether the resulting reader and cases samples are financially and logistically feasible. If not, then these three steps are repeated as needed, changing either the effect size or power in step 1 each time, until an acceptable combination of effect size, power, and reader and case samples sizes results. We also note that prior knowledge of the effect size should be taken into account when applicable. We illustrate these concepts below.

In Sec. 3.4, we showed how to compute the needed reader and case sample sizes for detecting an effect size of 0.05 with power = 0.80 and significance level = 0.05 based on the pilot data discussed in Sec. 3.1, but we did not say how the researcher chose the effect size of 0.05. Suppose that the researcher had chosen 0.05 because she considered it to represent the minimum clinically significant effect size. This approach has the advantage of providing adequate estimated power to detect any clinically meaningful effect size. Although the CI (−0.00036,0.088) from the pilot study contains values <0.05, because the researcher deems those values to be clinically negligible, there is no need to use a smaller specified effect size. On the other hand, if the upper CI bound had been <0.05 (e.g., 0.04), then there would appear to be no reason to plan another study since then we could conclude from the CI that the modality difference is clinically negligible.

In contrast, suppose that the researcher had considered 0.03 to represent the minimum clinically significant effect size but had sized the study to detect a 0.05 effect size because of financial or logistical limitations. Recalling that the researcher computed the necessary sample sizes to provide 80% power, it follows that estimated power is <0.80 for clinically significant effect sizes between 0.03 and 0.05. In addition, it seems quite possible that the true effect size could be between 0.03 and 0.05 because this range of values is roughly in the center of the effect-size CI computed from the pilot data. Thus, in this situation, we would recommend that the researcher try to obtain more resources so that a lower effect size could be detected with sufficient power.

A reviewer has noted that sometimes researchers will use an estimated effect size (e.g., from pilot data) for sizing a future study. This will result in the study being sufficiently powered for effects sizes at least as large as the estimate but not for effect sizes less than the estimate. Although we believe this may be a reasonable approach to use when the nature of the outcome makes it difficult to interpret effect size in a clinically meaningful way, in general, we recommend the approach discussed above that takes into account both clinical relevance and prior knowledge of effect size.

## 5 Other Designs

### 5.1 Design Descriptions

Thus far, we have only discussed the factorial study design, where each reader evaluates all cases under each test. However, the program can be used for sizing four other balanced study designs in addition to the factorial design. A brief description of these designs, with alternative names given in parentheses, is included below. For these models, μ denotes the fixed effect of test; all random effects are assumed to be normally distributed with zero means; and all random effects are assumed to be independent except for the error terms.

1. Factorial design (fully crossed design; paired-reader, paired-case design). Each reader evaluates all cases under each test. This is the most frequently used design and optimizes efficiency for a given number of readers and cases. The OR model for analyzing data collected using this design is given by model M1 (1).
2. Reader-nested-within-test split plot design (unpaired-reader, paired-case design). Cases undergo all tests, but each reader evaluates cases for only one of the tests. That is, the readers differ between the tests, with the number of readers the same for each test. This study design is natural when readers are trained to read under only one of the tests, or when each “test” level represents a descriptive characteristic (e.g., resident or radiologist) for each reader, as briefly discussed in Sec. 2.4. The OR model for analyzing data collected using this design is given by ${\hat{\theta }}_{ij}=\mu +\tau_{i}+R_{(i)j}+\epsilon_{ij}$, where $R_{i(j)}$ denotes the random effect of reader j nested in test i and $\epsilon_{ij}$ is the error term, having respective variances $\sigma_{R(C)}^{2}$ (reader-nested-within-test variance) and $\sigma_{\epsilon }^{2}$. The error terms have two possible covariances, ${\mathrm{Cov}}_{2}$ and ${\mathrm{Cov}}_{3}$, defined in the same way as for model M1. There is no ${\mathrm{Cov}}_{1}$ because each reader reads under only one test.
3. Case-nested-within-test split plot design (paired-reader, unpaired-case design). Each reader evaluates all of the cases, but each case is imaged under only one test, with equal numbers of cases imaged under each test. This design is needed when the diagnostic tests are mutually exclusive, for example, if they are invasive, administer a high radiation dose, or carry a risk of contrast reactions. The OR model for analyzing data collected using this design is the same as model M1 (1) with the additional constraints ${\mathrm{Cov}}_{1}={\mathrm{Cov}}_{3}=0$.
4. Case-nested-within-reader split plot design (paired-case per reader, paired-reader design). Each reader evaluates a different set of cases using all of the diagnostic tests. Compared to a factorial design, the advantage of this design is that typically the same power can be achieved with each reader interpreting fewer cases, but the disadvantage is that the total number of cases is higher. The OR model for analyzing data collected using this design is the same as model M1 (1) with the additional constraints ${\mathrm{Cov}}_{2}={\mathrm{Cov}}_{3}=0$.
5. Mixed split plot design (factorial-nested-within-group design). There are several groups (or blocks) of readers and cases such that each reader and each case belongs to only one group, and within each group all readers evaluate all cases under each test. Each group has the same numbers of readers and cases. If there is only one reader per block, then this design reduces to the case-nested-within-reader split plot design. The motivation for this study design is to reduce the number of reader interpretations for each reader, compared to the factorial study design, without requiring as many cases to be verified as the case-nested-within reader design. The OR model for analyzing data collected using this design is given by θ^ij=μ+τi+R(h)j+(τR)(h)ij+ϵhij, where $R_{h(j)}$ denotes the random effect of reader j nested in group h, ${\left(\tau R\right)}_{(h)ij}$ denotes the random test-by-reader interaction effect nested in group h, and $\epsilon_{hij}$ is the error term, having respective variances $\sigma_{R(G)}^{2}$ (reader-nested-within-group variance), $\sigma_{TR(G)}^{2}$ (test-by-reader interaction-nested-within-group variance), and $\sigma_{\epsilon }^{2}$. The error terms, ${\mathrm{Cov}}_{1}$, ${\mathrm{Cov}}_{2}$, and ${\mathrm{Cov}}_{3}$, are defined in the same way as for model M1, except that they are not defined between errors corresponding to different groups because the covariance of those errors is zero. (We note that this formulation differs slightly from that given by Hillis ^8^, which also adjusts for group.)

Note that designs 2 and 3 are not used to improve efficiency, but rather are needed for studies where readers are restricted to reading under only one of the tests or when diagnostic tests are mutually exclusive, which rules out using the factorial design. In contrast, designs 4 and 5 can be used in studies where the factorial design could also be used and are motivated by the need to reduce the number of reader interpretations per reader, although they will require more cases. See Ref. 25 for a discussion of designs 2, 3, and 4, and Refs. 26 and 27 for a discussion of design 5. Hillis^8^ provided rigorous derivations of the nonnull test statistics for all five designs, which are the basis for the sample size computations in the program.

### 5.2 Using Factorial-Model Parameter Inputs with Other Designs

The program is designed such that the parameter inputs for the factorial model can be used to compute sample size results for the other four study designs. Briefly, the relationships between the factorial model parameters and the parameters for the other designs are as follows. The design 3 and design 4 model parameters are the same as for the factorial model, except that ${\mathrm{Cov}}_{1}={\mathrm{Cov}}_{3}=0$ for design 3 and ${\mathrm{Cov}}_{2}={\mathrm{Cov}}_{3}=0$ for design 4. Thus their parameters are estimated by the corresponding factorial-model estimates. The design 2 model parameters include a reader-nested-within-test variance component, an error variance component, and ${\mathrm{Cov}}_{2}$ and ${\mathrm{Cov}}_{3}$. The last three of these parameters are estimated by the corresponding factorial-model estimates. Because the reader-nested-within-test variance has the same interpretation as the sum of the factorial-model reader and reader-by-test variance components, it is estimated by their sum. Finally, the design 5 model parameters include a reader-nested-within-group variance component, a test-by-reader interaction variance component nested within group, an error variance, and ${\mathrm{Cov}}_{2}$ and ${\mathrm{Cov}}_{3}$. The first two parameters have the same interpretations as the factorial reader and test-by-reader interaction variance components, respectively, and the last three parameters are the same as those for the factorial model; thus, these five parameters are estimated by the corresponding factorial-model estimates. For further discussion of these models, see Ref. 8.

### 5.3 Example: Comparing Sample Size Results for the Factorial and Reader-Nested-Within-Test Split-Plot Study Designs

In this example, we compute the number of cases and readers needed to achieve 0.8 power for a reader-nested-within-test study design, treating readers, and cases as random, using the parameter estimates obtained from the Van Dyke factorial study, shown in Fig. 1, as inputs. We then compare the results with those obtained in Sec. 3.4 for a factorial study.

**Fig. 14:**
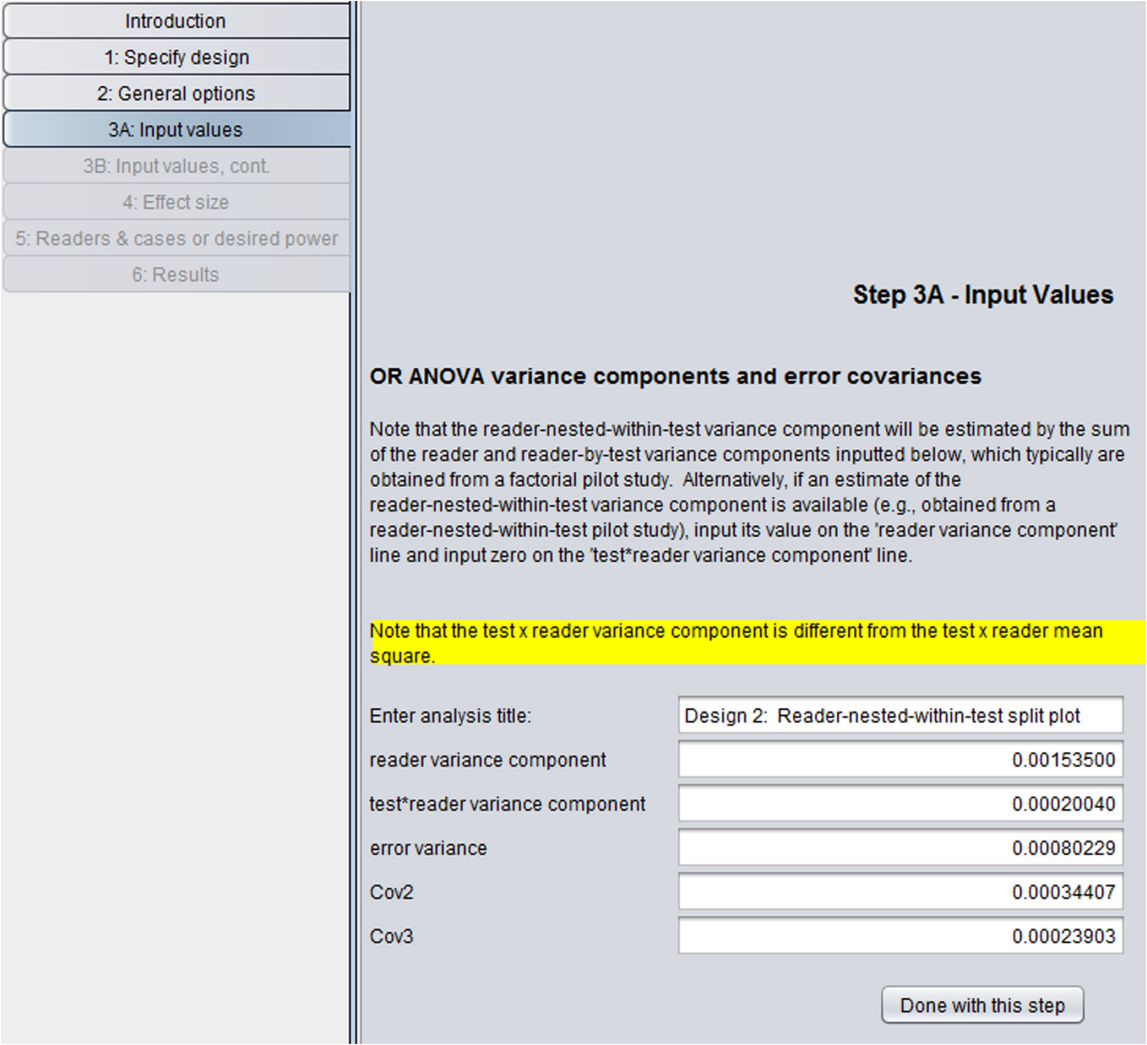
Step 3A for reader-nested-within-test split-plot study design.

In step 1 of the program, we indicate the second option, the reader-nested-within-test design. In step 2, we click on the same options as in Fig. 4 for the factorial design. Figure 14 shows our step 3A: input values” window inputs. Note that the requested inputs in step 3A are the same as for the factorial design, shown in Fig. 5, except that the reader variance is requested but not ${\mathrm{Cov}}_{1}$. We provide the same inputs in steps 3B, 4, and 5 as we did for the factorial design in Sec. 3.4.

**Fig. 15:**
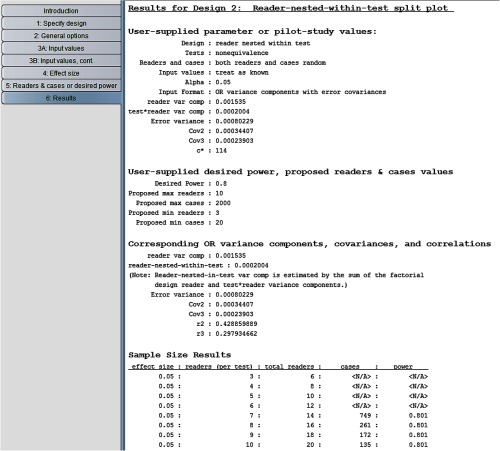
Results for reader-nested-within-test split-plot study design.

Results are shown in Fig. 15. Note that the second column shows the number of readers per test. We see that 10 readers in each of the two tests reading 135 cases results in 0.801 power, resulting in 2×10×135=2700 total readings. In contrast, we see from Fig. 9 that for the factorial model, 0.801 power is achieved with eight readers reading 134 cases under both tests, resulting in 2×8×134=2144 total readings. Thus, the factorial design is more efficient in the sense that it requires fewer total readings and fewer readers (8 versus 20) for approximately the same number of cases. However, as previously mentioned, if available readers are trained to read under only one of the tests, then the factorial design is not an option. More generally, several factors, including the training of the readers and availability and cost of both readers and cases, will enter into the decision of which study design is most suitable for a particular situation and research question.

## 6 Using Conjectured Parameter Estimates

Our opinion is that whenever possible, parameter inputs for sizing MRMC studies should be obtained from a pilot study or from previous studies that are similar to the planned study. However, in the absence of such studies, a researcher may want to use conjectured values, e.g., for computing initial ballpark sample size estimates for a study. Although a thorough discussion of how to determine conjectured parameter estimates is beyond the scope of this paper, in this section, we give a brief introduction to using conjectured inputs for sizing a factorial-design study having two tests. This section can be skimmed or skipped by readers only interested in using parameter estimates obtained from previous studies or pilot studies. However, we note that the discussion in this section is also helpful for interpreting the magnitude of the test-by-reader variance component and error correlation estimates obtained at the analysis stage.

For a given type of study, parameter values are affected by several factors, including the type and magnitude of the accuracy outcome (e.g., ROC AUC, sensitivity, specificity, etc.), type of disease, technology, training, and experience of the readers. Thus, there is no one set of conjectured values that are appropriate for every situation.

The needed OR parameter values for sizing a factorial design, using the notation in Fig. 1, are var(T*R), var(error), ${\mathrm{Cov}}_{1}$, and ${\mathrm{Cov}}_{2}-{\mathrm{Cov}}_{3}$. Here, we are assuming that the researcher wants to generalize to both the reader and case populations. In place of ${\mathrm{Cov}}_{1}$ and ${\mathrm{Cov}}_{2}-{\mathrm{Cov}}_{3}$, $r_{1}$ and $r_{2}-r_{3}$ can be used. Note that we do not need the individual values for ${\mathrm{Cov}}_{2}$ and ${\mathrm{Cov}}_{3}$ or $r_{2}$ and $r_{3}$ but rather only the difference. Also note that we do not need var(R). Larger values of var(T*R), var(error), and ${\mathrm{Cov}}_{2}-{\mathrm{Cov}}_{3}$ (or $r_{2}-r_{3}$), and smaller values of ${\mathrm{Cov}}_{1}$ (or $r_{1}$) result in more conservative sample size estimates, i.e., larger samples sizes for a given power.

It can be shown (see Sec. 11 Appendix C) that var(T*R), the test-by-reader variance component, is equal to half of the variance of the test1 – test2 differences in the true reader accuracies, where a reader’s “true reader accuracy” can be conceptually thought of as the average accuracy outcome if the reader was to read many randomly selected case samples. (More precisely, a fixed reader’s true accuracy is the expected value of the accuracy outcome for a randomly selected sample of cases.) Thus, var(T*R) is a measure of between-test reader performance variability.

For typical reader accuracy outcomes, var(T*R) will not depend on or only slightly depend on the reader or case sample sizes, or on the normal-to-abnormal case ratio. For example, the expected value (true accuracy) for the empirical AUC is Pr(Y>X)+.5Pr(Y=X), where Y and X are ratings given to a pair of randomly chosen abnormal and normal cases, respectively; for continuous ratings, this value does not depend on the reader or case sample sizes, or on the normal-to-abnormal case ratio. Note that the true reader accuracies are not observed and will have less variability than the observed accuracies computed from the data, which include random measurement error due to the random selection of cases and within-reader variability.

Table 1 shows the relationship between the range of the middle 95% of the true-accuracy difference distribution and the corresponding var(T*R) value. For example, if for the population of readers the middle 95% of the test1 – test2 true-accuracy differences range between −0.02 and 0.08, resulting in a middle 95% range of 0.10, then var(T*R)=0.00033. The results in Table 1 follow from the fact that the middle 95% range is approximately equal to $3.92\times \sqrt{\mathrm{var}(T*R)}$, which follows from the normality of the true accuracies, implied by the OR model.

**Table 1:** Relationship between middle 95% range of test1-minus-test2 true-accuracy differences across the reader population and test-by-reader interaction variance, denoted by var(T*R). These results are for any reader-performance outcome.

95% range	var(T*R)
0.01	0.00000
0.02	0.00001
0.03	0.00003
0.04	0.00005
0.05	0.00008
0.06	0.00012
0.07	0.00016
0.08	0.00021
0.09	0.00026
0.10	0.00033
0.11	0.00039
0.12	0.00047
0.13	0.00055
0.14	0.00064
0.15	0.00073

Typically, conjectured values for the error correlations, $r_{1}$, $r_{2}$, and $r_{3}$, are used instead of the error covariances, ${\mathrm{Cov}}_{1}$, ${\mathrm{Cov}}_{2}$, and ${\mathrm{Cov}}_{3}$, because they have been shown in simulations to be relatively stable across different case and reader sample sizes when rating data are generated from the same probabilistic statistical model.^28^ In contrast, the covariances are dependent on the case sample sizes. The correlation $r_{1}$ is the within-reader between-test correlation of accuracy measurement errors for a fixed reader when reading random samples of cases. Thus, it is a measure of similarity of within-reader measurement errors for the two tests. The difference $r_{2}-r_{3}$ describes, for two fixed readers, similarity in between-reader correlation of accuracy measurement errors within one test ($r_{2}$) versus between two different tests ($r_{3}$). When tests 1 and 2 are very similar in nature, we expect $r_{2}-r_{3}$ to be close to zero. For 20 MRMC studies reported by Rockette et al.^29^ with ROC AUC as the outcome, values of $r_{1}$ ranged from 0.35 to 0.59 (median=0.48) and values of $r_{2}-r_{3}$ ranged from −0.0.0196 to 0.0139. By comparison, $r_{1}$ and $r_{2}-r_{3}$ were estimated to be 0.43 and 0.429−0.298=0.131, respectively, for the Van Dyke study (Fig. 1). It should be noted, however, that the 20 studies were not independently performed. Instead, the 20 studies are based on subsets of data from two original studies. Each of the extracted studies is based on the same 529 cases and use one of two unique sets of six readers.

When the outcome is the ROC AUC, the error variance is often estimated using the following equation, proposed by Obuchowski:^30^

$$\mathrm{var}(\text{error})=\frac{1}{n_{1}}0.0099\;\exp(-a^{2}/2)[(5a^{2}+8)+(a^{2}+8)/R],$$ (5)

where $a=\sqrt{2}\phi^{-1}(\mathrm{AUC})$, AUC is the average of the test1 and test2 AUCs, $\phi^{-1}$ is the inverse of the cumulative normal distribution function, $n_{1}$ is the number of abnormal cases, $n_{0}$ is the number of normal cases, and R is the normal-to-abnormal case ratio, $n_{0}/n_{1}$. Obuchowski and McClish^31^ found this estimator to work reasonably well for ordinal and continuous rating data when either parametric or nonparametric estimates of the AUC are used. Note that an assumption about the AUC must be made to compute Eq. (5) and that smaller values of AUC produce larger values of var(error).

### 6.1 Examples

#### 6.1.1 Comparison of conjectured and Van Dyke error variances

In this example, we compare the conjectured error variance obtained from Eq. (5), using the mean of the two test reader-averaged AUC estimates and the case sample sizes from the Van Dyke study (Fig. 1), with the error variance estimated from the Van Dyke data. For the Van Dyke study, the mean AUC is (0.897+0.941)/2=0.92, $n_{0}=69$, $n_{1}=45$, and R=69/44. Using these values with Eq. (5) yields an error variance of 0.00109, which compares with the data estimate of 0.00080 given in Fig. 1; thus, the conjectured value is 36% higher and hence will produce more conservative sizing results.

#### 6.1.2 Using conjectured inputs with the program

In this example, we illustrate using the program to size a factorial study using conjectured values for all of the parameters. For the proposed study, there will be two tests and ROC AUC will be the reader-performance outcome. Suppose the researcher wants to obtain a conservative ballpark idea of the needed sample size to obtain 80% power to detect an effect size of 0.06 for a nonequivalence test, treating both readers and cases as random. She believes the mean of the two test AUCs is no lower than 0.85. She plans to choose experienced readers for the study and thus expects that variability among the readers’ comparisons of the two tests will be low, with the most of the test1 – test2 true AUC differences being within a range no larger than 0.06; thus based on Table 1 she uses 0.0001 for a conservative conjectured value of var(T*R). Because the two tests are rather similar, she believes that $r_{2}-r_{3}$ will not exceed 0.05, and thus uses 0.05 as a conservative conjectured value. She has no idea what to expect about $r_{1}$, so she chooses a conservative conjectured value of 0.35, the minimum value from Ref. 29. Finally, she wants to use equal numbers of normal and abnormal cases.

Inputting the values $n_{1}=100$, R=1, and AUC=0.85 into Eq. (5) results in var(error)=0.000977. (Note that R=1 because the researcher wants equal numbers of normal and abnormal cases.) The value of $n_{1}$ is arbitrary: any value of $n_{1}$ can be used, as long as the inputted value for “total number of cases (c*)” in step 3B is equal to $n_{0}+n_{1}$, or equivalently, $n_{1}(1+R)$, where $n_{0}$ and R (or $n_{0}$ and $n_{1}$) are the values used in Eq. (5).

**Fig. 16:**
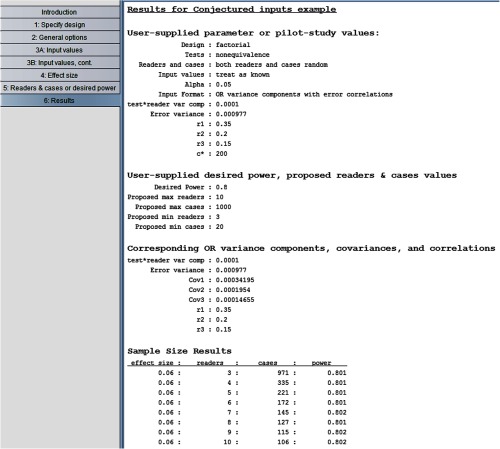
Results using conjectured inputs.

Using the program with conjectured inputs proceeds the same as when using pilot-study inputs except for two differences. First, as noted above, the value for $c^{*}$ in step 3B must correspond to the values used in Eq. (5), resulting in $c^{*}=200$ for our example. Second, the values inputted for $r_{2}$ and $r_{3}$ can be any values between 0 and 1, as long as $r_{2}-r_{3}$ equals the conjectured difference. Thus, for our example, where the conjectured difference is 0.05, inputting $r_{2}=0.20$, $r_{3}=0.15$ or $r_{2}=0.30$, $r_{3}=0.25$ will give the same results. Results using these conjectured values are shown in Fig. 16. We see, e.g., that 172 cases are needed with six readers.

### 6.2 Other Remarks

In this section. we have provided a brief introduction to using conjectured values. For further examples, see Ref. 32 [pp. 220–225] and Obuchowski and Hillis.^33^ However, a problem with these two references is that the equation used for estimating the test-by-reader interaction variance component is positively biased, as noted by Hillis et al. [18, p 134]. It would be especially helpful for deciding on conjectured estimates if researchers would list all of the model parameter estimates when they publish an MRMC study, which presently is rarely done. In addition, it would be helpful to have available more studies that give the parameter estimates for several MRMC studies, similar to the study by Rockette.^29^ Finally, we note that Rockette study also includes estimates for the test-by-reader interaction variance component, but we have not utilized these in our discussion because they appear to have been computed incorrectly, as discussed in Sec. 12 Appendix D.

## 7 Sample-Size Computation Methodology for Factorial Test-by-Reader-by-Case Study Design

In this section, we discuss the methodology underlying the software. Throughout, we assume rating data have been collected using a balanced test-by-reader-by-case factorial study design, where each of r readers assigns a likelihood-of-disease rating to each case using t=2 tests. This is the most frequently used study design for multireader diagnostic imaging studies. In this paper, we limit our discussion to this design; methodology for the other four designs will be discussed separately.

For the reader mainly interested in using the software, this section can be skimmed or skipped.

### 7.1 Models

In this section, we discuss the analysis models that correspond to the three inference situations discussed in Sec. 2.4.

#### 7.1.1 Random readers and random cases model

Let θ^ij denote the AUC estimate (or more generally, a reader-performance outcome), which has been computed from the likelihood-of-disease ratings assigned by reader j using test i to each case. For analyzing these reader performance outcomes, OR^2^ proposed a test-by-reader factorial ANOVA model where the error terms are correlated to account for correlation resulting from each reader evaluating the same cases. With i=1,…,t and j=1,…,r, their model is given as

Model M1:  θ^ij=μ+τi+Rj+(τR)ij+ϵij, (6)

where μ is a fixed intercept term, $\tau_{i}$ denotes the fixed effect of test i, $R_{j}$ denotes the random effect of reader j, ${\left(\tau R\right)}_{ij}$ denotes the random test-by-reader interaction, and $\epsilon_{ij}$ is the error term. Model M1 treats both reader and case as random factors, and thus conclusions generalize to both the reader and case populations. We note that model M1 given by Eq. (6) is the same as model M1 given by Eq. (1) in Sec. 3.2, but we repeat some of the description provided in Sec. 3.2 to make this section easier to read.

The $R_{j}$ and ${\left(\tau R\right)}_{ij}$ are assumed to be mutually independent and normally distributed with zero means and respective variances $\sigma_{R}^{2}$ and $\sigma_{TR}^{2}$. The $\epsilon_{ij}$ are assumed to be normally distributed with mean zero and variance $\sigma_{\epsilon }^{2}$ and are assumed independent of the $R_{j}$ and ${\left(\tau R\right)}_{ij}$. Equicovariance of the errors between readers and tests is assumed, resulting in three possible covariances:

$$\mathrm{Cov}(\epsilon_{ij},\epsilon_{i^{\prime }j^{\prime }})=\{\begin{array}{ll} {\mathrm{Cov}}_{1} & i\neq i^{\prime },j=j^{\prime }(\text{different test},\text{same reader})\\ {\mathrm{Cov}}_{2} & i=i^{\prime },j\neq j^{\prime }(\text{same test},\text{different reader})\\ {\mathrm{Cov}}_{3} & i\neq i^{\prime },j\neq j^{\prime }(\text{different test},\text{different reader}) \end{array}.$$ (7)

We assume

$${\mathrm{Cov}}_{1}\geq {\mathrm{Cov}}_{3},\;{\mathrm{Cov}}_{2}\geq {\mathrm{Cov}}_{3}\;\text{and}\;{\mathrm{Cov}}_{3}\geq 0$$ (8)

as recommended by Hillis.^8^ It follows from Eq. (6) that $\sigma_{\epsilon }^{2}$, ${\mathrm{Cov}}_{1}$, ${\mathrm{Cov}}_{2}$, and ${\mathrm{Cov}}_{3}$ are also the variance and corresponding covariances of the AUC estimates, treating readers as fixed. Thus, $\sigma_{\epsilon }^{2}$, ${\mathrm{Cov}}_{1}$, ${\mathrm{Cov}}_{2}$, and ${\mathrm{Cov}}_{3}$ are typically estimated using fixed-reader methods such as the jackknife,^18^ bootstrap,^19^ or the method of DeLong et al.^15^ (DeLong’s method is only for empirical AUC.) Model M1 can alternatively be described with population correlations

$$r_{i}={\mathrm{Cov}}_{i}/\sigma_{\epsilon }^{2},\;i=1,2,3$$ (9)

instead of the covariances, i.e., with ${\mathrm{Cov}}_{i}$ replaced by $r_{i}\sigma_{\epsilon }^{2}$, i=1,2,3.

The $\epsilon_{ij}$ are interpreted as AUC measurement error attributable to the random selection of cases, and to within-reader variability attributable to variation in how a fixed reader interprets the same images on different occasions that are separated by a memory washout period. Accordingly, OR^2^ partition the error variance into two components

$$\sigma_{\epsilon }^{2}=\sigma_{c}^{2}+\sigma_{w}^{2},$$ (10)

where $\sigma_{c}^{2}$ denotes variability attributable to cases and $\sigma_{w}^{2}$ denotes within-reader variability. It follows, as suggested by Hillis et al.,^21^ that we can write the error term as the sum

$$\epsilon_{ij}=u_{ij}+w_{ij},$$

where $u_{ij}$ denotes the random effect of cases; $w_{ij}$ denotes the random within-reader effect; $u_{ij}$ and $w_{ij}$ are normally distributed with zero means and with $\mathrm{var}(u_{ij})=\sigma_{c}^{2}$, $\mathrm{var}(w_{ij})=\sigma_{w}^{2}$; the $w_{ij}$ are mutually independent and are independent of the $u_{ij}$; and the $u_{ij}$ are correlated and have the same covariance structure as the $\epsilon_{ij}$, i.e.,

$$\mathrm{Cov}(u_{ij},u_{i^{\prime }j^{\prime }})=\mathrm{Cov}(\epsilon_{ij},\epsilon_{i^{\prime }j^{\prime }}).$$ (11)

It follows that model M1 can be written in the form:

Model M1 (alternative form):  θ^ij=μ+τi+Rj+(τR)ij+uij+wij. (12)

OR^2^ expressed the covariances in the form ${\mathrm{Cov}}_{i}=\sigma_{c}^{2}r_{i}$, i=1,2,3, where $r_{i}$ is defined by $r_{i}={\mathrm{Cov}}_{i}/\sigma_{c}^{2}$, which is the correlation of the $\left(u_{ij},u_{ij^{\prime }}\right)$ pair corresponding to ${\mathrm{Cov}}_{i}$. We use the correlation definitions given by Eq. (9) because they are used by current software, as it is not possible to estimate $\sigma_{c}^{2}$ or $\sigma_{w}^{2}$ without replications.

Although $\sigma_{c}^{2}$ and $\sigma_{w}^{2}$ cannot be estimated without replications, we show in Sec. 9 Appendix A that

$$\sigma_{w}^{2}\leq \sigma_{\epsilon }^{2}-{\mathrm{Cov}}_{1}-({\mathrm{Cov}}_{2}-{\mathrm{Cov}}_{3}).$$ (13)

In Sec. 7.4, we will utilize Eq. (13) for estimating sample size for the fixed-cases model.

#### 7.1.2 Fixed-readers model

For an analysis for which conclusions apply only to the readers in the study, we treat the reader and test-by-reader effects as fixed in model M1, as given by Eq. (6). This results in the fixed-readers model

Model M2:  θ^ij=μ+τi+Rj+(τR)ij+ϵij, (14)

where $R_{j}$ denotes the fixed effect of reader j and ${\left(\tau R\right)}_{ij}$ denotes the fixed test-by-reader interaction. The $\epsilon_{ij}$ are assumed to be normally distributed with mean zero and variance $\sigma_{\epsilon }^{2}$ and to be equicovariant as defined by Eq. (7). This model has been discussed in Refs. 2 and 6. Without loss of generality, we impose the following constraints on model M2:

$$\overset{r}{\underset{j=1}{\sum }}R_{j}=\overset{2}{\underset{i=1}{\sum }}{\left(\tau R\right)}_{ij}=\overset{r}{\underset{j=1}{\sum }}{\left(\tau R\right)}_{ij}=0.$$ (15)

#### 7.1.3 Fixed-cases model

For an analysis that treats readers as random and cases as fixed, we set $\sigma_{c}^{2}=0$ in model M1, as given by Eq. (12), resulting in the fixed-cases model:

Model M3:  θ^ij=μ+τi+Rj+(τR)ij+wij, (16)

where the $R_{j}$, ${\left(\tau R\right)}_{ij}$, and $w_{ij}$ are assumed to be mutually independent and normally distributed with zero means and respective variances $\sigma_{R}^{2}$, $\sigma_{TR}^{2}$, and $\sigma_{w}^{2}$. Note that unlike models M1 and M2 for which the error terms are equicovariant, we assume independence of the $w_{ij}$ error terms in model M3.

### 7.2 Nonequivalence Test Hypotheses and Test Statistics

Our software computes the needed sample sizes for comparing t=2 tests. For the nonequivalence test, the null hypothesis states that the two tests are equivalent and the alternative hypothesis states that they are not equivalent, with equivalence defined in terms of the expected reader performance outcomes. In this section, we describe these hypotheses for each of the three models and present the corresponding test statistics.

#### 7.2.1 Random readers and random cases analysis

It follows from model M1, as specified by Eq. (6), that the expected reader performance outcome for test i is $E({\hat{\theta }}_{ij})=\mu +\tau_{i}$. This is the expected performance outcome for a randomly selected reader reading a randomly selected case sample. The null hypothesis of equivalence states that the two tests have the same expected reader performance outcomes, i.e., H0:E(θ^1j)=E(θ^2j), or equivalently, $H_{0}:\mu +\tau_{1}=\mu +\tau_{2}\Leftrightarrow \tau_{1}=\tau_{2}$. The alternative hypothesis of nonequivalence states that they are not equal, i.e., H1:E(θ^1j)≠E(θ^2j), or equivalently, $H_{1}:\tau_{1}\neq \tau_{2}$.

The test statistic for testing these hypotheses is

F=MS(T)MS(T*R)+r max(Cov^2−Cov^3,0), (17)

where Cov^2 and Cov^3 are estimates for ${\mathrm{Cov}}_{2}$ and ${\mathrm{Cov}}_{3}$, and MS(T) and MS(T*R) are the test and test-by-reader mean squares computed from the reader performance outcomes θ^ij, i=1,2, j=1,…,r. Specifically, MS(T)=r∑i=12(θ^i·−θ^··)2/(t−1) and MS(T*R)=∑i=12∑j=1r(θ^ij−θ^i·−θ^·j+θ^··)2/[(t−1)(r−1)], where θ^1· and ${\hat{\theta }}_{2\cdot }$ denote the mean of the reader AUC estimates for test 1 and test 2, respectively; i.e., θ^i·=1r∑j=1rθ^ij, i=1,2. Note that t−1=1 since we assume t=2 tests. Letting $F_{p;{\mathrm{df}}_{1},{\mathrm{df}}_{2}}$ denote the p(100)th percentile of an F distribution with numerator and denominator degrees of freedom ${\mathrm{df}}_{1}$ and ${\mathrm{df}}_{2}$, respectively, for significance level α the null hypothesis is rejected if $F>F_{1-\alpha ;{\mathrm{df}}_{1},{\mathrm{df}}_{2}}$, where ${\mathrm{df}}_{1}=t-1=1$ and

df2=[MS(T*R)+r max(Cov^2−Cov^3,0)]2[MS(T*R)]2/[(t−1)(r−1)]. (18)

Hillis^6^ derived Eq. (18) and showed that it resulted in improved performance of the original OR method.

#### 7.2.2 Fixed-readers analysis

It follows from model M2, as specified by Eq. (14), that the expected reader performance outcome for test i and fixed reader j reading a randomly selected case sample is given as

E(θ^ij)=μ+τi+Rj+(τR)ij. (19)

The null hypothesis of equivalence states that the two tests have the same mean expected reader performance outcomes, where the mean is computed across the study readers. It follows from Eqs. (15) and (19) that for test i, the mean of the expected performance outcomes for the r fixed study readers is given by

1r∑j=1rE(θ^ij)=1r∑j=1r[μ+τi+Rj+(τR)ij]=μ+τi.

Thus, the null hypothesis of equivalence is H0:1r∑j=1rE(θ^1j)=1r∑j=1rE(θ^2j), or equivalently, $H_{0}:\tau_{1}=\tau_{2}$, and the alternative hypothesis of nonequivalence is H1:1r∑j=1rE(θ^1j)≠1r∑j=1rE(θ^2j), or equivalently, $H_{1}:\tau_{1}\neq \tau_{2}$. Although in terms of the $\tau_{i}$ parameters, these hypotheses are identical to those of model M1, their interpretation is different. For the fixed-readers model, the hypotheses imply the tests are equal or not equal in terms of the averages of the expected performance outcomes for the specific readers in the study, whereas for model M1, the hypotheses imply the tests are equal or not equal in terms of the expected performance outcome for a randomly chosen reader.

The test statistic for testing these hypotheses is

χ2=(t−1)MS(T)σϵ2−Cov^1+(r−1)max(Cov^2−Cov^3,0). (20)

Letting $\chi_{p;{\mathrm{df}}_{1}}^{2}$ denote the p(100)th percentile of a chi-squared distribution with ${\mathrm{df}}_{1}$ degrees of freedom, for significance level α the null hypothesis is rejected if $\chi^{2}>\chi_{1-\alpha ;{\mathrm{df}}_{1}}^{2}$ where ${\mathrm{df}}_{1}=t-1=1$. Test statistic Eq. (20) has been discussed in Refs. 2, 5, and 6. Briefly, the OR model implies that the numerator of the right side of Eq. (20), (t−1)MS(T), has a chi-squared null distribution with t−1=1  degree of freedom when divided by $E[{\mathrm{MS}(T)|H}_{0}]$, the expected value of MS(T) given the null hypothesis is true. The denominator is an estimate of $E[{\mathrm{MS}(T)|H}_{0}]$. Thus, if there is a moderate number of cases, resulting in relatively precise error variance and covariance estimates, then the right side of Eq. (20) will have an approximate chi-squared null distribution with t−1=1  degree of freedom.

#### 7.2.3 Fixed-cases analysis

It follows from model M3, as specified by Eq. (16), that the expected reader performance outcome for test i is given by $E({\hat{\theta }}_{ij})=\mu +\tau_{i}$. This is the expected reader performance value for a randomly selected reader, restricted to evaluating only the study cases.

The null hypothesis of equivalence states that the two tests have the same expected reader performance outcomes, i.e., H0:E(θ^1j)=E(θ^2j), or equivalently, $H_{0}:\mu +\tau_{1}=\mu +\tau_{2}\Leftrightarrow \tau_{1}=\tau_{2}$. The alternative hypothesis of nonequivalence states that they are not equal, i.e., $H_{1}:E({\hat{\theta }}_{1j})\neq E({\hat{\theta }}_{2j})$, or equivalently, $H_{1}:\tau_{1}\neq \tau_{2}$. Although these hypotheses are mathematically the same as for model M1, their interpretation is different. For this model, the hypotheses imply that the tests are equal or not equal in terms of the expected value for a randomly chosen reader reading only the study cases, rather than a randomly selected sample of cases.

Model M3 is a conventional test-by-reader ANOVA model with independent errors, where reader is a random factor and test is a fixed factor. This is the same as a repeated measures ANOVA model where test is the repeated measures factor, i.e., each reader provides a reader-performance outcome under each test. For two tests, this analysis is equivalent to a paired t test performed on the reader-performance outcomes. The conventional ANOVA test statistic is given as

$$F=\frac{\mathrm{MS}(T)}{\mathrm{MS}(T*R)}.$$ (21)

For significance level α, the null hypothesis is rejected if $F>F_{1-\alpha ;t-1,(t-1)(r-1)}$.

### 7.3 Power Computation

#### 7.3.1 Overview

To compute power, we must specify the nonnull distribution of the test statistic, i.e., the distribution if $H_{0}$ is not true. For model M1, the approximate nonnull distribution has been derived by Hillis.^6^^,^^8^ For models M2 and M3, the derivations of the approximate nonnull distributions are straightforward and are included in Sec. 10 Appendix B. For models M1 and M3, the nonnull distribution of the F test statistic is a noncentral F distribution; for model M2, the nonnull distribution of the $\chi^{2}$ test statistic is a noncentral chi-squared distribution. Thus, specification of the nonnull distribution requires specification of the noncentrality parameter and the degrees of freedom in terms of the model parameters. We assume t=2 tests for all computations.

Let $F_{{\mathrm{df}}_{1},{\mathrm{df}}_{2};\lambda }$ denote a random variable having a noncentral F distribution with degrees of freedom ${\mathrm{df}}_{1}$ and ${\mathrm{df}}_{2}$ and noncentrality parameter λ. If ${\mathrm{df}}_{1}$, ${\mathrm{df}}_{2}$, and λ correctly specify the nonnull distribution of the F statistic Eq. (17) or Eq. (21) for model M1 or M3, respectively, then for significance level α the power of the test is given as

$$\mathrm{Pr}(F_{{\mathrm{df}}_{1},{\mathrm{df}}_{2};\lambda }>F_{1-\alpha ;{\mathrm{df}}_{1},{\mathrm{df}}_{2}}),$$ (22)

which is the probability that the F test statistic exceeds the critical value $F_{1-\alpha ;{\mathrm{df}}_{1},{\mathrm{df}}_{2}}$, i.e., the probability that the null hypothesis is rejected. Similarly, let $\chi_{{\mathrm{df}}_{1};\lambda }^{2}$ denote a random variable with a noncentral chi-squared distribution with degrees of freedom ${\mathrm{df}}_{1}$ and noncentrality parameter λ. If ${\mathrm{df}}_{1}$ and λ correctly specify the nonnull distribution of the $\chi^{2}$ test statistic Eq. (20) for model M2, then the power of the test is given as

$$\mathrm{Pr}(\chi_{{\mathrm{df}}_{1};\lambda }^{2}>\chi_{1-\alpha ;{\mathrm{df}}_{1}}^{2}).$$ (23)

#### 7.3.2 Algorithm for determining nonnull-distribution parameter formulas

The nonnull-distribution parameter equations can be determined from the test statistic equations in the following way. With t=2, for each of the three test statistics discussed in Sec. 7.2, the numerator is MS(T), which can be written in the form

MS(T)=r2(θ^1·−θ^2·)2. (24)

The noncentrality parameter can be obtained by replacing ${\hat{\theta }}_{1\cdot }$ and θ^2· in Eq. (24) by their expected values and dividing by the expected value of the denominator of the test statistic after replacing ${\mathrm{Cov}}_{1}$, ${\mathrm{Cov}}_{2}$, and ${\mathrm{Cov}}_{3}$ estimates by their true values.

That is, the noncentrality parameter, denoted by λ, is given by

$$\lambda =\frac{\frac{r}{2}d^{2}}{E(\mathrm{denom})},$$ (25)

where the “effect size” d is the difference of the expected mean AUCs

d=E(θ^2·)−E(θ^1·), (26)

and “denom” denotes the denominator of the test statistic after the covariance estimates have been replaced by their true values.

For model M1, ${\mathrm{df}}_{1}=t-1=1$ and ${\mathrm{df}}_{2}$ can be obtained from the test-statistic ${\mathrm{df}}_{2}$ equations by replacing covariance estimates by their true values and mean squares by their expected values. For models M2 and M3, the degrees of freedom do not depend on model parameters and are given by ${\mathrm{df}}_{1}=t-1=1$ for model M2, and by ${\mathrm{df}}_{1}=t-1=1$ and ${\mathrm{df}}_{2}=(t-1)(r-1)$ for model M3. (Note that there is no ${\mathrm{df}}_{2}$ for model M2 since its test statistic has a chi-squared nonnull distribution.)

Below we illustrate using the algorithm to obtain the equations for the noncentrality parameters for all three models and for ${\mathrm{df}}_{2}$ for model M1.

#### 7.3.3 Random readers and random cases

For model M1, the denominator is MS(T*R)+r max(Cov^2−Cov^3,0). Assuming the covariance constraints given by Eq. (8), replacing covariance estimates by their true values yields

$$\mathrm{denom}=\mathrm{MS}(T*R)+r({\mathrm{Cov}}_{2}-{\mathrm{Cov}}_{3}).$$ (27)

Hillis^6^^,^^8^ shows that

$$E[\mathrm{MS}(T*R)]=\sigma_{\tau R}^{2}+\sigma_{\epsilon }^{2}-{\mathrm{Cov}}_{1}-{\mathrm{Cov}}_{2}+{\mathrm{Cov}}_{3}.$$ (28)

It follows from Eqs. (27) and (28) that

$$E(\mathrm{denom})=E[\mathrm{MS}(T*R)]+r({\mathrm{Cov}}_{2}-{\mathrm{Cov}}_{3})\;=\sigma_{TR}^{2}+\sigma_{\epsilon }^{2}-{\mathrm{Cov}}_{1}+(r-1)({\mathrm{Cov}}_{2}-{\mathrm{Cov}}_{3}).$$

It follows from Eq. (25) that the noncentrality parameter is given by

$$\lambda =\frac{rd^{2}/2}{\sigma_{TR}^{2}+\sigma_{\epsilon }^{2}-{\mathrm{Cov}}_{1}+(r-1)({\mathrm{Cov}}_{2}-{\mathrm{Cov}}_{3})}.$$ (29)

To write the degrees of freedom ${\mathrm{df}}_{2}$ in terms of the model parameters, in Eq. (18) we replace MS(T*R) by its expected value [Eq. (28)] and estimated covariances by their true values; i.e., taking into account the constraints given by Eq. (8), we replace max(Cov^2−Cov^3,0) by ${\mathrm{Cov}}_{2}-{\mathrm{Cov}}_{3}$. This results in

$${\mathrm{df}}_{2}=\frac{{\left[\sigma_{TR}^{2}+\sigma_{\epsilon }^{2}-{\mathrm{Cov}}_{1}+(r-1)({\mathrm{Cov}}_{2}-{\mathrm{Cov}}_{3})\right]}^{2}}{{\left(\sigma_{TR}^{2}+\sigma_{\epsilon }^{2}-{\mathrm{Cov}}_{1}-{\mathrm{Cov}}_{2}+{\mathrm{Cov}}_{3}\right)}^{2}/[(t-1)(r-1)]}.$$ (30)

#### 7.3.4 Fixed-readers model

For model M2, $\mathrm{denom}=\sigma_{\epsilon }^{2}-{\mathrm{Cov}}_{1}+(r-1)({\mathrm{Cov}}_{2}-{\mathrm{Cov}}_{3})$ results after replacing covariances estimates in the denominator of Eq. (20) by their true values. Because there are no random quantities in denom, it follows that E(denom)=denom. From Eq. (25), it follows that the noncentrality parameter is given by

$$\lambda =\frac{rd^{2}/2}{\sigma_{\epsilon }^{2}-{\mathrm{Cov}}_{1}+(r-1)({\mathrm{Cov}}_{2}-{\mathrm{Cov}}_{3})}.$$ (31)

As noted above, ${\mathrm{df}}_{1}=t-1=1$.

#### 7.3.5 Fixed-cases model

For model M3, the denominator is MS(T*R). It follows that denom = MS(T*R) because there are no covariance estimates. For this conventional repeated-measure ANOVA model, it is well known that $E[\mathrm{MS}(T*R)]=\sigma_{TR}^{2}+\sigma_{w}^{2}$. Hence, $E(\mathrm{denom})=\sigma_{TR}^{2}+\sigma_{w}^{2}$ and

$$\lambda =\frac{rd^{2}/2}{\sigma_{TR}^{2}+\sigma_{w}^{2}}.$$ (32)

As discussed in Sec. 7.3.2, ${\mathrm{df}}_{1}=t-1=1$ and ${\mathrm{df}}_{2}=(t-1)(r-1)=r-1$.

The nonnull-distribution noncentrality parameters and the degrees of freedom for all three models are presented in Table 2.

**Table 2:** Nonnull-distribution parameters and their corresponding estimates for the nonequivalence test for the factorial design. Models M1 and M3 each have an approximate noncentral F nonnull distribution with noncentrality parameter λ and degrees of freedom ${\mathrm{df}}_{1}=t-1=1$ and ${\mathrm{df}}_{2}$. Model M2 has an approximate noncentral, chi-squared nonull distribution with noncentrality parameter λ, and degrees of freedom ${\mathrm{df}}_{1}=t-1=1$. Notes: d = effect size as defined by Eq. (26); t = number of tests, with t=2; r = number of readers; $c^{*}$ = number of cases in the pilot study from which parameter estimates were computed, or the number of cases corresponding to the error variance if parameter values are conjectured; c = number of cases in planned study; ${\hat{\sigma }}_{TR}^{2}$, ${\hat{\sigma }}_{\epsilon }^{2}$, ${\hat{\mathrm{Cov}}}_{1}$, ${\hat{\mathrm{Cov}}}_{2}$, and ${\hat{\mathrm{Cov}}}_{3}$, are OR estimates from a factorial-design pilot study with $c^{*}$ cases, or are conjectured values; NA = not applicable.

Nonnull-distribution parameters:		
Model	λ	${\mathrm{df}}_{2}$
M1	$\frac{rd^{2}/2}{\sigma_{TR}^{2}+\sigma_{\epsilon }^{2}-{\mathrm{Cov}}_{1}+(r-1)({\mathrm{Cov}}_{2}-{\mathrm{Cov}}_{3})}$	$\frac{{\left[\sigma_{TR}^{2}+\sigma_{\epsilon }^{2}-{\mathrm{Cov}}_{1}+(r-1)({\mathrm{Cov}}_{2}-{\mathrm{Cov}}_{3})\right]}^{2}}{{\left(\sigma_{\tau R}^{2}+\sigma_{\epsilon }^{2}-{\mathrm{Cov}}_{1}-{\mathrm{Cov}}_{2}+{\mathrm{Cov}}_{3}\right)}^{2}/[(t-1)(r-1)]}$
M2	$\frac{rd^{2}/2}{\sigma_{\epsilon }^{2}-{\mathrm{Cov}}_{1}+(r-1)({\mathrm{Cov}}_{2}-{\mathrm{Cov}}_{3})}$	NA
M3	$\frac{rd^{2}/2}{\sigma_{TR}^{2}+\sigma_{w}^{2}}$	(t−1)(r−1)=r−1
Nonnull-distribution parameter estimates:		
Model	$\hat{\lambda }$	${\hat{\mathrm{df}}}_{2}$
M1	rd2/2σ^TR2+c*c[σ^ϵ2−Cov^1+(r−1)max(Cov^2−Cov^3,0)]	{σ^TR2+c*c[σ^ϵ2−Cov^1+(r−1)max(Cov^2−Cov^3,0)]}2{σ^TR2+c*c[σ^ϵ2−Cov^1−max(Cov^2−Cov^3,0)]}2/[(t−1)(r−1)]
M2	rd2/2c*c[σ^ϵ2−Cov^1+(r−1)max(Cov^2−Cov^3,0)]	NA
M3	rd2/2σ^TR2+c*c[σ^ϵ2−Cov^1−max(Cov^2−Cov^3,0)]	(t−1)(r−1)=r−1

#### 7.3.6 Interpretation of the effect size d

The interpretation of the effect size d depends on the interpretation of E(θ^i·), i=1,2 for each of the three models. It follows from the definitions of the models that d can be interpreted as follows:

1. For model M1, d is the difference in the test 1 and test 2 expected reader performance outcomes for a randomly selected reader evaluating a randomly selected case sample.
2. For model M2, d is the difference of the test 1 and test 2 averages of the expected reader outcomes for the r fixed study readers, when evaluating a randomly selected case sample.
3. For model M3, d is the difference in the test 1 and test 2 expected reader performance outcomes for a randomly selected reader evaluating the study cases.

Note that because models M1 and M3 treat readers as a random sample, each reader has the same expected performance outcome for a given test. In contrast, model M2 treats readers as fixed, with each fixed reader having a (generally) different expected performance outcome for a given test, resulting in $E({\hat{\theta }}_{i\cdot })$ being the average of these r different fixed-reader expectations.

### 7.4 Parameter Estimation

Power estimation requires estimates for the parameters $\sigma_{TR}^{2},\sigma_{\epsilon }^{2}$, ${\mathrm{Cov}}_{1}$, ${\mathrm{Cov}}_{2}-{\mathrm{Cov}}_{3}$ (or $r_{1}$ and $r_{2}-r_{3}$) n-order to estimate the noncentrality parameters and degrees of freedom. As previously discussed, estimates for these parameters can be obtained from analysis of pilot data using freely available software, or they can be conjectured.

The parameter values depend on the number of cases and the diseased-to-nondiseased case ratio. Following Hillis, Berbaum, and Obuchowski,^21^ we assume for a fixed case ratio that the error variance and covariances are directly proportional to case sample size while $\sigma_{R}^{2}$ and $\sigma_{TR}^{2}$ remain constant for different case sizes and diseased-to-nondiseased ratios. These assumptions were shown^21^ to approximately hold in simulations. Thus, for power computations, the pilot-study estimates of the error variance and covariances must be adjusted to account for differences in the pilot data and power computation sample sizes. Specifically, letting $c^{*}$ denote the number of pilot-study cases that were evaluated by each reader, these estimates are multiplied by a factor of $c^{*}/c$ when computing power for c cases. If parameter values are conjectured rather than estimated from pilot data, then $c^{*}$ is the number of cases corresponding to the conjectured error variance. In contrast, the values of $\sigma_{R}^{2}$ and $\sigma_{TR}^{2}$ used in the power computation for various reader and sample sizes are the estimates obtained from pilot data.

For example, when computing power for model M1 for a study with c cases and r readers, Eqs. (29) and (30) are modified accordingly, resulting in noncentrality parameter and degrees of freedom estimates

$$\hat{\lambda }=\frac{rd^{2}/2}{{\hat{\sigma }}_{TR}^{2}+\frac{c^{*}}{c}[{\hat{\sigma }}_{\epsilon }^{2}-{\hat{\mathrm{Cov}}}_{1}+(r-1)\max({\hat{\mathrm{Cov}}}_{2}-{\hat{\mathrm{Cov}}}_{3},0)]},$$

and

df^2={σ^TR2+c*c[σ^ϵ2−Cov^1+(r−1)max(Cov^2−Cov^3,0)]}2{σ^TR2+c*c[σ^ϵ2−Cov^1−max(Cov^2−Cov^3,0)]}2/[(t−1)(r−1)],

where σ^TR2, σ^ϵ2, Cov^1, Cov^2, and ${\hat{\mathrm{Cov}}}_{3}$ denote pilot-study estimates and $c^{*}$ denotes the number of cases in the pilot study. Here, we have partially imposed the constraints given by Eq. (8) by using the term $\max({\hat{\mathrm{Cov}}}_{2}-{\hat{\mathrm{Cov}}}_{3},0)$ in place of ${\mathrm{Cov}}_{2}-{\mathrm{Cov}}_{3}$.

Similarly, for model M2, we modify Eq. (31) accordingly, resulting in the noncentrality parameter estimate

λ^=rd2/2c*c[σ^ϵ2−Cov^1+(r−1)max(Cov^2−Cov^3,0)].

For model M3, there typically will not be an estimate of $\sigma_{w}^{2}$ available from pilot data because estimation requires replicated data. Thus, we propose using

σ^w2=c*c[σ^ϵ2−Cov^1−max(Cov^2−Cov^3,0)] (33)

as a conservative estimate for $\sigma_{w}^{2}$, which is justified by Eq. (13). Combining this estimate with Eq. (32) yields

λ^=rd2/2σ^TR2+c*c[σ^ϵ2−Cov^1−max(Cov^2−Cov^3,0)]

as our noncentrality estimate.

Estimates for the null-distribution noncentrality parameters and degrees of freedom for all three models are presented in Table 2. Correlation estimates can alternatively be used instead of covariance estimates by replacing ${\hat{\mathrm{Cov}}}_{i}$ by riσ^ϵ2, i=1,2,3 in the noncentrality and degrees-of-freedom estimate formulas.

### 7.5 Example

Table 3 illustrates the use of Table 2 equations and Eqs. (22) and (23) for computing power for detecting a 0.05 modality difference in AUC for a study with seven readers and 148 cases, based on the Van Dyke data parameter estimates from Fig. 1, with alpha=0.05. Note that the power of 0.802 for model M1 agrees with the power computed by the program for seven readers and 148 cases, included under “sample size results” in Fig. 9. As it is typical, the power estimates for models M2 (0.899) and M3 (0.945) exceed that for model M1 (0.802); these power estimates for models M2 and M3 can be obtained from the program by specifying “power for specified reader and case sample sizes” in step 2 and then requesting power for seven readers and 148 cases in step 5.

**Table 3:** Power computation example. This table illustrates use of Table 2 equations for computing power for detecting a 0.05 difference in AUC for a study design having seven readers and 148 cases, based on the Van Dyke parameter estimates from Fig. 1, with alpha=0.05.

Model M1:
λ^=rd2/2σ^TR2+cc*[σ^ϵ2−Cov^1+(r−1)max(Cov^2−Cov^3,0)]=7(0.05)2/20.00020040+114148[0.00080229−0.00034661+(7−1)(0.00034407−0.00023903)]=8.439
df^2={σ^TR2+cc*[σ^ϵ2−Cov^1+(r−1)max(Cov^2−Cov^3,0)]}2{σ^TR2+cc*[σ^ϵ2−Cov^1−max(Cov^2−Cov^3,0)]}2/[(t−1)(r−1)]={0.00020040+114148[0.00080229−0.00034661+(7−1)(0.00034407−0.00023903)]}2{0.00020040+114148[0.00080229−0.00034661−(0.00034407−0.00023903)]}2/(7−1)=29.140
Power=Pr(Fdf1,df^2;λ^>F1−α;df1,df^2)=Pr(F1,29.14;8.439>F0.95;1,29.14=4.18122)=0.802
Model M2:
λ^=rd2/2c*c[σ^ϵ2−Cov^1+(r−1)max(Cov^2−Cov^3,0)]=7(0.05)2/2114148[0.00080229−0.00034661+(7−1)(0.00034407−0.00023903)]=10.461
Power=Pr(χdf1;λ^2>χ1−α;df12)=Pr(χ1;10.4612>χ0.95;12=3.8416)=0.899
Model M3:
λ^=rd2/2σ^TR2+c*c[σ^ϵ2−Cov^1−max(Cov^2−Cov^3,0)]=7(0.05)2/20.00020040+114148[0.00080229−0.00034661−(0.00034407−0.00023903)]=18.598
df^2=(t−1)(r−1)=7−1=6
Power=Pr(Fdf1,df^2;λ^>F1−α;df1,df^2)=Pr(F1,6;18.598>F0.95;1,6=5.9874)=0.945

### 7.6 Noninferiority Test Hypotheses

We assume that a higher value of the reader-performance measure indicates better performance. Letting S and N denote “standard” and “new” tests, the hypotheses for a noninferiority test^34^^–^^40^ are given as

H0:E(θ^Sj)−E(θ^Nj)≥M (N is inferior to S),H1:E(θ^Sj)−E(θ^Nj)<M (N is not inferior to S), (34)

where M>0 is the noninferiority margin. The null hypothesis states that the reader performance outcome for the standard test exceeds that of the new test by at least M. If $H_{0}$ is true, the new test is considered to be inferior to the standard test. The alternative hypothesis states that the standard test may result in higher reader performance but by less than M. If $H_{1}$ is true, the new test is considered to be noninferior to the standard test. We define the effect size for the noninferiority test by

dinf=E(θ^Nj)−E(θ^Sj). (35)

Note that $H_{1}$ implies $d_{\inf}>-M$.

A one-sided hypothesis test can be performed at significance level α by computing a 100(1−α)% upper confidence bound (UCB) for $E({\hat{\theta }}_{Sj})-E({\hat{\theta }}_{Nj})$ and rejecting $H_{0}$ if UCB is less than M. Alternatively, equivalent results can be obtained by computing a two-sided 100(1−2α)% CI for $E({\hat{\theta }}_{Sj})-E({\hat{\theta }}_{Nj})$ and rejecting $H_{0}$ if the interval lies entirely to the left of M. For typical power values of interest (e.g., ≥0.7), power is for practical purposes equal to power for a two-sided nonequivalence test with significance level 2α and effect size E(θ^Nj)−E(θ^Sj)+M=dinf+M, where $d_{\inf}$ is given by Eq. (35). This is the approach used in the program. Specifically, power computations for the inferiority test with significance level α are performed as for the nonequivalence test using the nonnull distribution equations in Table 2, but with the significance level set to 2α and with d in column 1 of in Table 2 replaced by $d_{\inf}+M$.

In practice, often a significance level of 0.025 is used for the noninferiority test so that the decision rule will be based on a 95% two-sided CI, which is consistent with guidelines^41^ provided by the US Food and Drug Administration and which provides consistency between significance testing and subsequent estimation using 95% CIs.^38^ See Ref. 34 for a more detailed discussion of noninferiority hypotheses testing for multireader ROC studies.

## 8 Discussion

The software “Multireader sample size program for diagnostic studies” is a useful tool for sizing radiologic diagnostic studies because of its ease of use and options for study designs, types of hypotheses, and input and output formats; furthermore, it is applicable to parametric and nonparametric reader-performance outcomes, which include outcomes from ROC, FROC, LROC, and ROI analyses. We illustrated use of the program, followed by a discussion of the underlying statistical methodology. To keep the paper at a reasonable length while at the same time satisfying the needs of most researchers, methodology was discussed only for the most commonly used study design, the factorial design. However, separately we will discuss methodology and provide more examples for the other four designs included in the program.

Although sample-size methodology for the factorial study design for the OR and DBM methods has been discussed by Hillis et al.,^21^ their discussion is limited to model M1, which treats both readers and cases as random. A main contribution of this paper is that it also includes a discussion of sample-size methodology for models M2 and M3, which treat readers or cases as fixed, respectively. Another contribution is the algorithm for determining nonnull-distribution parameter equations, provided in Sec. 7.3.2. This algorithm makes it easy to write down the nonnull distributions directly from the test statistic equations and can be used for all of the study designs included in the program.

Power and sample size methodology for multireader diagnostic studies can be based on methods of analysis other than the OR method. For the situation where the reader-performance outcome of interest is the empirical AUC, Gallas et al.^42^^,^^43^ have developed an often-used analysis method that utilizes the fact that the empirical AUC is a U-statistic, which enables its variance to be expressed in closed form as a linear combination of product moments of functions of the ratings. An advantage of this method over the OR method is that it is straightforward to size future studies for which the abormal-to-normal case ratio differs from that of the pilot study. Software for this method is freely available.^44^ A limitation of this approach is that the reader-performance outcome must be a U-stastistic, such as the empirical AUC; in contrast, the OR method is applicable to all reader-performance outcomes. Although there have been several bootstrapping approaches proposed for multireader diagnostic studies (see Ref. 43 for a comparison and a discussion of bias problems), they are rarely used.

Finally, our intention has been to create a self-contained program that prompts the user for needed inputs and requires minimal statistical understanding. We welcome and appreciate questions and feedback related to using the program, as well as suggestions for improving it.

## 9 Appendix A: Derivation of Eq. (13)

For this proof, notation and definitions are the same as in Sec. 7.1. It follows from Eqs. (7) and (11) that

$$\mathrm{var}(u_{11}-u_{12}-u_{21}+u_{22})=4(\sigma_{c}^{2}-{\mathrm{Cov}}_{1}-{\mathrm{Cov}}_{2}+{\mathrm{Cov}}_{3}).$$

Because $\mathrm{var}(u_{11}-u_{12}-u_{21}+u_{22})\geq 0$, it follows that $4(\sigma_{c}^{2}-{\mathrm{Cov}}_{1}-{\mathrm{Cov}}_{2}+{\mathrm{Cov}}_{3})\geq 0$. Hence

$$\sigma_{c}^{2}\geq {\mathrm{Cov}}_{1}+{\mathrm{Cov}}_{2}-{\mathrm{Cov}}_{3}.$$ (36)

Equation (13) follows from Eqs. (10) and (36).

## 10 Appendix B: Nonnull Distribution Derivations for Models M2 and M3

In this section, we derive the nonnull distributions for models M2 and M3. Notation and definitions are the same as in Sec. 7. Recall that for both models the nonequivalence-test hypotheses are $H_{0}:\tau_{1}=\tau_{2}$ versus $H_{1}:\tau_{1}\neq \tau_{2}$.

### 10.1 Derivation of Nonnull F Distribution for Model M3

Result 1: For model M3 with t=2 tests, the nonnull distribution of the F-test statistic Eq. (21) has an F distribution with degrees of freedom ${\mathrm{df}}_{1}=t-1=1$ and ${\mathrm{df}}_{2}=(t-1)(r-1)$ and noncentrality parameter

$$\lambda =\frac{rd^{2}/2}{\sigma_{TR}^{2}+\sigma_{w}^{2}}.$$ (37)

Proof. Let ${\mathrm{df}}_{1}=t-1$ and ${\mathrm{df}}_{2}=(t-1)(r-1)$. Model M3 is a conventional ANOVA model with independent errors. It is well known^45^ that MS(T) and MS(T*R) are independent, with

$$\frac{{\mathrm{df}}_{1}\mathrm{MS}(T)}{E[\mathrm{MS}(T)|H_{0}]}∼\chi_{{\mathrm{df}}_{1};\lambda }^{2}\;\text{and}\;\frac{{\mathrm{df}}_{2}\mathrm{MS}(T*R)}{E[\mathrm{MS}(T*R)]}∼\chi_{{\mathrm{df}}_{2}}^{2},$$ (38)

where

$$E[\mathrm{MS}(T)|H_{0}]=E[\mathrm{MS}(T*R)]=\sigma_{TR}^{2}+\sigma_{w}^{2},$$ (39)

λ=df1MS(T)|θ^ij=E(θ^ij)E[MS(T)|H0], (40)

“∼” stands for “has the same distribution as,” $\chi_{{\mathrm{df}}_{1};\lambda }^{2}$ denotes a random variable with a noncentral chi-squared distribution with degrees of freedom ${\mathrm{df}}_{1}$ and noncentrality parameter λ, and $\chi_{{\mathrm{df}}_{2}}^{2}$ denotes a random variable with a central chi-squared distribution with degrees of freedom ${\mathrm{df}}_{2}$. These results do not require t=2.

From the independence of MS(T) and MS(T*R), Eqs. (21), (38), and (39) it follows that

$$F=\frac{\frac{\mathrm{MS}(T)}{E[\mathrm{MS}(T)|H_{0}]}}{\frac{\mathrm{MS}(T*R)}{E[\mathrm{MS}(T*R)]}}∼\frac{\chi_{{\mathrm{df}}_{1};\lambda }^{2}/{\mathrm{df}}_{1}}{\chi_{{\mathrm{df}}_{2}}^{2}/{\mathrm{df}}_{2}},$$

where the numerator and denominator of F are independent. It follows that F has a noncentral F distribution with degrees of freedom ${\mathrm{df}}_{1}=t-1$ and ${\mathrm{df}}_{2}=(t-1)(r-1)$ and noncentrality parameter λ, defined by Eq. (40).

To complete the proof we need to show for t=2 that λ, as given by Eq. (40), can be equivalently expressed in the form given by Eq. (37). For t=2, it follows from Eqs. (24) and (26) that

MS(T)|θ^ij=E(θ^ij)=r2[E(θ^1·)−E(θ^2·)]2=r2d2. (41)

From Eqs. (39), (40), and (41), it follows that

$$\lambda =\frac{rd^{2}/2}{\sigma_{TR}^{2}+\sigma_{w}^{2}}.$$

### 10.2 Derivation of Approximate Nonnull F Distribution for Model M2

*Result* 2: For model M2 with t=2, the nonnull distribution of the $\chi^{2}$ test statistic Eq. (20) has an approximate noncentral chi-squared distribution with degrees of freedom ${\mathrm{df}}_{1}=t-1=1$ and noncentrality parameter

$$\lambda =\frac{rd^{2}/2}{\sigma_{\epsilon }^{2}-{\mathrm{Cov}}_{1}+(r-1)({\mathrm{Cov}}_{2}-{\mathrm{Cov}}_{3})}.$$

Proof. Let t=2. We approximate the nonnull distribution of the $\chi^{2}$ test statistic Eq. (20) by deriving the nonnull distribution of

$${\overset{˜}{\chi }}^{2}=\frac{\left(t-1)\mathrm{MS}(T\right)}{\sigma_{\epsilon }^{2}-{\mathrm{Cov}}_{1}+(r-1)({\mathrm{Cov}}_{2}-{\mathrm{Cov}}_{3})},$$ (42)

which is similar to Eq. (20), except that covariance estimates have been replaced by true values.

Using Eq. (24) we can write

χ˜2=(θ^1·−θ^2.)22r[σϵ2−Cov1+(r−1)(Cov2−Cov3)]. (43)

It is straightforward to show that (θ^1·−θ^2·)∼N{−d,2r[σϵ2−Cov1+(r−1)(Cov2−Cov3)]}, and hence

(θ^1·−θ^2.)2r[σϵ2−Cov1+(r−1)(Cov2−Cov3)]∼N{−d2r[σϵ2−Cov1+(r−1)(Cov2−Cov3)],1}. (44)

Using the well-known result that if X∼N(μ,1) then $X^{2}∼\chi_{1;\mu^{2}}^{2}$, it follows from Eqs. (43)–(44) that ${\overset{˜}{\chi }}^{2}∼\chi_{{\mathrm{df}}_{1};\lambda }^{2}$, where

$$\lambda =\frac{rd^{2}/2}{\sigma_{\epsilon }^{2}-{\mathrm{Cov}}_{1}+(r-1)({\mathrm{Cov}}_{2}-{\mathrm{Cov}}_{3})}.$$

Because the $\chi^{2}$ test statistic Eq. (20) uses covariance estimates that usually are estimated relatively precisely because typically the number of cases is at least 50, we consider the null distribution of ${\tilde{\chi }}^{2}$ to be an approximation to the nonnull distribution of $\chi^{2}$.

## 11 Appendix C: Relationship Between Test-by-Reader Variance and Variance of Test1-Test2 Differences in True Reader Accuracies

For the OR model, given by Eq. 6, for test i and fixed reader j we define the true reader accuracy by

$$\theta_{ij}=\mu +\tau_{i}+R_{j}+{\left(\tau R\right)}_{ij}.$$

Note that $\theta_{ij}$ is equal to ${\hat{\theta }}_{ij}$ with the measurement error $\varepsilon_{ij}$ subtracted. Statistically, $\theta_{ij}$ is the expected value of ${\hat{\theta }}_{ij}$ for fixed reader j and test i across randomly selected case samples. It follows from the assumptions of the OR model (now treating both readers and cases as random) that

$$\sigma_{TR}^{2}=0.5\mathrm{var}(\theta_{1j}-\theta_{2j}),$$

i.e., the test-by-reader interaction variance component is equal to half of the variance of the test1-test2 differences in the true reader accuracies.

We note that this result has previously been given by Hillis,^28^ who refers to $\theta_{ij}$ as the reader-specific expected accuracy.

## 12 Appendix D: Apparent Incorrect Computation of var(T*R) Estimates in Rockette et al. Paper

The test-by-reader variance component estimates in the paper by Rockette et al.^29^ were computed using the DBM method, which, as previously mentioned, is equivalent to a special case of the OR method. The DBM and OR model test-by-reader interaction variance components have been shown^5^ to be equal and hence have the same interpretation. However, 14 of the 20 Var(T*R) reported values reported in the Rockette et al. paper exceed 0.125. This is problematic because, making the reasonable assumption that the true reader AUCs are between 0.5 and 1.0 (and hence the between-test differences range from −0.5 to 0.5), it follows that the largest possible variance for the between-test true AUC differences is 0.25, which implies (by the Sec. 11 Appendix C result) that the test-by-reader interaction variance component cannot exceed 0.25/2=0.125.

Rockette et al. described how they computed this variance component:

We also estimated the modality-by-case and modality-by-reader interaction by using the general procedure proposed by Dorfman, Berbaum, and Metz (4) [1992] for our four studies on resolution, brightness, and compression. To accomplish this, we obtained the 529 estimates of areas under the ROC curves associated with eliminating one case for each reader. The corresponding pseudovalues were then computed for all cases and used to conduct a mixed-model analysis of variance with readers and cases used as random effects and modality as a fixed effect.

We now explain their computations in more detail. Let ${\hat{\theta }}_{ij}$ denote the empirical AUC estimate for modality i and reader j, and let ${\hat{\theta }}_{ij(k)}$ denote the empirical AUC estimate for modality i and reader j when data for case k are omitted from the computation. Pseudovalues are defined for each possible modality-reader-case combination by Yijk=cθ^ij−(c−1)θ^ij(k), where c is the number of cases. The mixed-model analysis that they refer to assumes the model proposed by Dorfman et al.,^3^ known as the DBM model, for which the outcome is $Y_{ijk}$, modality is a fixed effect, reader and case are random effects, and modality-by-reader, modality-by-case, reader-by-case, and modality-by-reader-by-case interactions are included in the model as random effects.

Because the DBM software available at the time that the Rockette et al. paper was published did not output an estimate of var(T*R), it is reasonable to assume that they computed the estimate directly from the relevant mean squares. The conventional unbiased estimate of the modality-by-reader interaction, var(T*R), is given by [MS(T*R)−MS(T*R*C)]/c, where MS(T*R) and MS(T*R*C) are the test-by-reader and test-by-reader-by-case mean squares computed from the pseudovalues. We also note that this estimate can be deduced from Table 1 provided by Dorfman et al.,^3^ which includes the expected mean squares written in terms of the variance components. We suspect that the authors forgot to divide by c=529, because doing so results in var(T*R) estimates between 0.00013 and 0.00087, which is a more typical range.
